# Unc-51 Like Kinase 3 (ULK3) is essential for autophagy and cell survival in multiple myeloma

**DOI:** 10.21203/rs.3.rs-7160521/v1

**Published:** 2025-08-12

**Authors:** Marilena Tauro, Tao Li, Praneeth R. Sudalagunta, Mark Meads, Rafael Renatino Canevarolo, Niveditha Nerlakanti, Raghunandan R. Alugubelli, Harshani R. Lawrence, Steven Gunawan, Mohammad Ayaz, Pradeep Nareddy, Sang Young Yun, Gemma Shay, Kathy Yang, Timothy H. Tran, Ryan T. Bishop, Mostafa M. Nasr, Nicholas N.J. Lawrence, Ernst Schönbrunn, John L. Cleveland, Ariosto S. Silva, Kenneth H. Shain, Conor C. Lynch

**Affiliations:** 1 Department of Tumor Microenvironment & Metastasis, H. Lee Moffitt Cancer Center and Research Institute; Tampa, FL, USA.; 2 Department of Metabolism and Physiology, H. Lee Moffitt Cancer Center and Research Institute; Tampa, FL, USA.; 3 Cancer Biology Ph.D. Program, University of South Florida; Tampa, FL, USA; 4 Department of Malignant Hematology, H. Lee Moffitt Cancer Center & Research Institute; Tampa, FL, USA.; 5 Drug Discovery Program, H. Lee Moffitt Cancer Center and Research Institute; Tampa, FL, USA; 6 Chemical Biology Core, H. Lee Moffitt Cancer Center and Research Institute; Tampa, FL, USA

## Abstract

Despite the availability of effective therapies such as proteasome inhibitors, multiple myeloma (MM) patients relapse with refractory disease. To identify new therapeutic targets, we assessed RNA sequencing data from CD138^+^ MM patient cells (*n* = 813) across disease stages and found that an autophagy gene signature, and particularly ULK3 expression, was strongly associated with disease progression. Functional studies revealed that ULK3 contributes to MM cell survival as part of the ULK-ATG13-FIP200 complex. We generated inhibitors (SG3–014/MA9–060) with nanomolar potency and confirmed their binding mode through co-crystallization with ULK3. *In vivo*, ULK3 inhibition reduced MM burden, improved survival, and protected against cancer-induced bone disease. MA9–060 also restored sensitivity to proteasome inhibitors in resistant MM cells. This synergy was validated *ex vivo* in patient samples, especially those with high *ULK3* expression. These findings indicate a new role for ULK3-mediated autophagy in cancer and suggest that ULK3 inhibition is an effective treatment strategy for both newly diagnosed and refractory MM disease.

## INTRODUCTION

Multiple myeloma (MM) is an incurable bone marrow malignancy that causes bone destruction [1–3]. While approved therapies like proteasome inhibitors (PIs) [4], CAR-T and Bispecific T-cell Engagers (BiTEs)[5] have improved survival, relapse is inevitable, highlighting the need for new treatments. To discover new therapeutic targets, we leveraged RNA-Seq datasets generated at Moffitt from CD138^+^ MM cells derived from the bone marrow aspirates of newly diagnosed, relapsed/refractory patients, as well as those with monoclonal gammopathy of undetermined significance (MGUS) and smoldering multiple myeloma (SMOL) (*n* = 813). Bioinformatic analysis confirmed that genes regulating autophagy correlate with disease stage, highlighting the role of autophagy in supporting MM cell survival [6]. Ongoing clinical trials with chloroquine (CQ) show promise but are hindered by off-target toxicities and incomplete understanding of the underlying mechanisms of action [7–9]. This underscores the need for selective inhibitors targeting key autophagy regulators.

Induction of autophagy involves dephosphorylation of the ULK complex (ULK1, ATG13, FIP200) when mTOR is suppressed upon nutrient starvation [10, 11]. ULK1 inhibitors have been developed due to its role in autophagy and expression in various cancers [12–14]. However, our human patient datasets showed no link between ULK1 expression and MM disease stage. In contrast, compared to other ULK kinase family members, ULK3 expression strongly correlated with MM progression. Although little is known about ULK3 in cancer, studies on its Drosophila ortholog, ADUK, suggest that it plays a key role in autophagy initiation upon chemotherapy-induced stress [15]. These findings have significant implications for anticancer treatment. Interestingly, ULK3 has a known involvement in cellular abscission, a process regulated by autophagy-related components like Beclin-1 [16]. ULK3 has also been implicated in oncogenic pathways including its interaction with GLI2 [17], which regulates anti-apoptotic proteins such as BCL-2 [18], linking autophagy to cell survival in certain cancers [19, 20]. Given our own findings and recent literature [21], we evaluated further whether ULK3 played a causal role in MM autophagy and disease progression.

## RESULTS

### ULK3 expression correlates with MM progression

**Figure 1.**
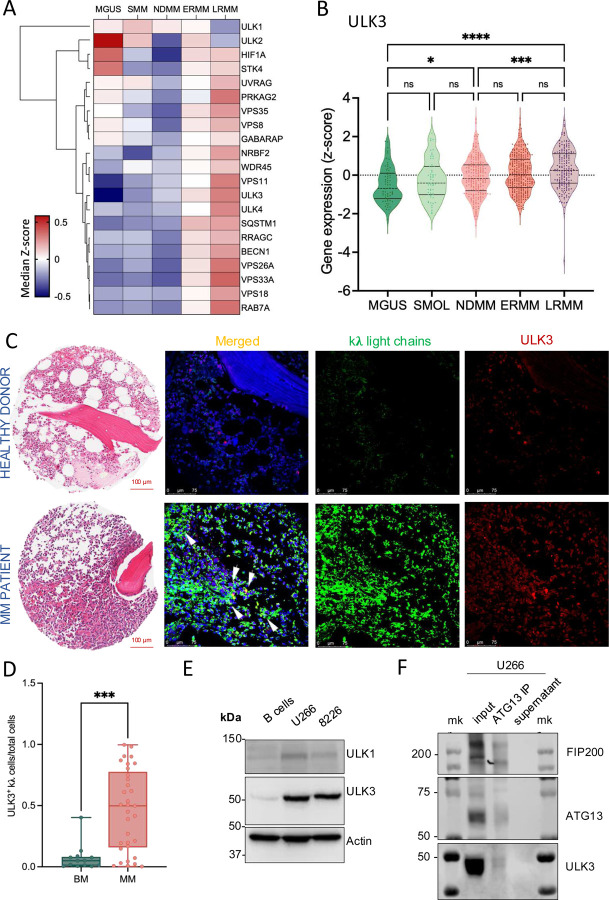
Elevated ULK3 expression is a hallmark of MM disease progression and connotes worse outcome. **A.** Heatmap representation of the average-linkage hierarchical clustering (correlation-based distance) and the expression levels (measured as median Z-score values) of single genes in the autophagy signature across MM disease stage cohorts (MGUS; n = 64), Smoldering (SMOL; n = 57), Newly Diagnosed (NDMM; n = 207), Early Relapsed (ERMM; n = 303) and Late Relapsed MM (LRMM; n = 182). **B**. ULK3 expression in Moffitt MM patients divided in disease stage cohorts. Representation as violin plots. Asterisks denote significance (Significance: (****) P<0.0001, (***) P<0.001, (**) P<0.01, (*) P<0.05, One-way ANOVA followed by Tukey’s test). **C**. Representative images of a healthy donor’s bone marrow biopsy core (top) and multiple myeloma patient (bottom) from TCGA program Tissue Micro Arrays (hematoxylin and eosin stain H&E stained, scale bar; 100 μm) and corresponding confocal microscopy images of the kλ light chains^+^/ULK3 immunofluorescence staining (anti-human kλ light chain Dako F0198, F0199 in green), Alexa Fluor647 anti-ULK3 [EPR4888] (ab310161 in red), DAPI nuclear staining in blue. IgG controls were used (Millipore Gt X Hu Ig k and λ CAT# AP-502, AP-506). Acquisition by Zeiss 488/647. Arrows represent colocalization of ULK3 protein and anti-human kλ light chain positive cells, resulting in yellow staining. **D**. Immunofluorescence quantification of ULK3^+^ kλ cells/total cells in biopsies of normal bone marrow (n = 15) and MM bone marrow (n = 34) (unpaired t test, p = 0.0002). **E**. Immunoblot of ULK1 and ULK3 protein levels in primary isolated CD19^+ve^ human B-cells, and MM cell lines (U266 and 8266). Actin was used as a loading control. **F.** Immunoblotting analysis of the ATG13-FIP200-ULK protein complex upon ATG13 pulldown in parental U266 MM cell lysates. Molecular marker showed here as mk.

Understanding cell survival programs and/or signaling pathways in MM progression and relapse [22] can reveal therapeutic targets. We leveraged RNA-seq data from CD138^+^ MM cells obtained from Moffitt patients classified as MGUS (*n* = 64), smoldering (SMOL; *n* = 57), newly diagnosed (NDMM; treatment naïve, *n* = 207), early relapsed/refractory (ERMM; 1–3 lines of therapy, *n* = 303) and late relapsed/refractory (LRMM; >3 lines of therapy *n* = 182) [23]. A set of autophagy-associated genes (identified by Enrichr pathway analysis, KEGG 2021 Human, Reactome 2022; ontologies, GO biological process 2023) was evaluated in our database and correlated with MM progression stages (Fig. 1A). Average-linkage hierarchical clustering and expression levels of single autophagy genes across MM stages were also assessed [24].

Dissecting our patient dataset, we observed that ULK3 expression correlated with disease stage (Fig. 1B), unlike other ULK kinase family members including *ULK1,* which has been well studied in autophagy, and *ULK2* (Supplementary Fig. 1A, B). We did however note that *ULK4* expression was significantly higher in relapsed patients (Supplementary Fig. 1C). We validated the increased expression of *ULK3* compared to precursor states in independent data sets (GSE5900 combined with GSE2658; Supplementary Fig. 1D–G) [25, 26] However, the lack of LRMM patients did not allow for independent validation of our *ULK4* findings. Of note, other autophagy components (for example Beclin-1, RAB7A, SQSTM1) also increased expression with disease stage but given the potential of ULK3 as an initiator of the program, combined with the fact that ULK4 is a pseudo kinase, prompted us to interrogate ULK3 further.

We observed that ULK3 levels were significantly elevated in MM patient samples compared to kλ light chain positive plasma cells in healthy bone marrow specimens (Fig. 1C, D). Elevated expression of ULK3 was also present in MM cell lines compared to CD19^+^ B-cells isolated from healthy donors (Fig. 1E, Supplementary Fig. 1H–I). Immunoprecipitation and proteomic analysis revealed ULK3 can bind to the ATG13:FIP200 autophagy subcomplex, indicating a potentially new role for ULK3 in initiating autophagy (Fig. 1F and Supplementary Fig. 2).

### ULK3 is an essential regulator of autophagy in MM

**Figure 2.**
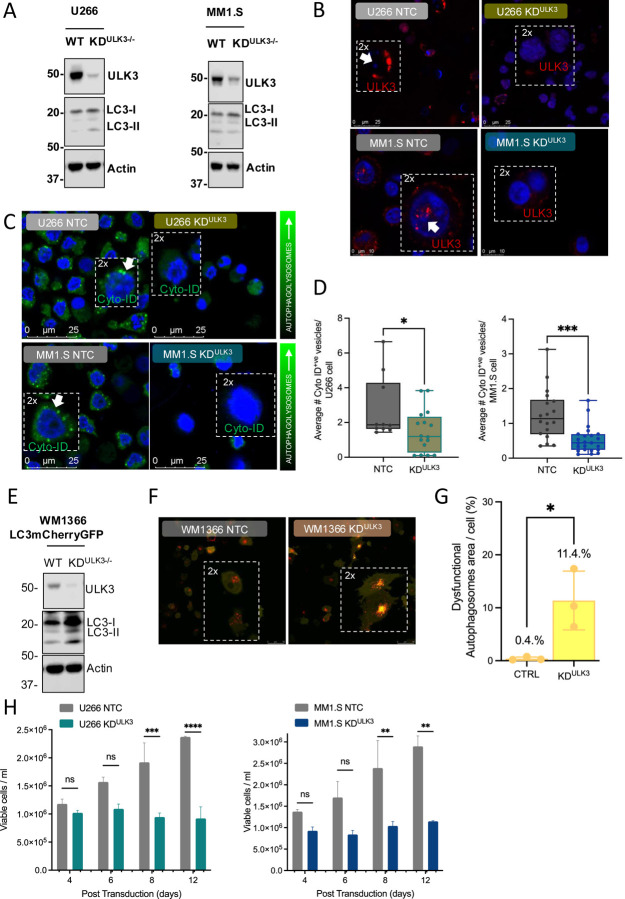
ULK3 regulates autophagy and survival of MM cell lines. **A**. Immunoblotting of wild-type (WT) vs ULK3 knockdown using CRISPR gene editing (KD^ULK3−/−^) in U266 and MM1.S MM cells (24 hours from transfection). **B**. Representative images of ULK3 immunofluorescent staining (Anti-ULK3 antibody EPR4888) in U266 NTC vs. U266 KD^ULK3^ (scale 1–10 μm) and MM1.S NTC vs. MM1.S KD^ULK3^ (scale 1–10 μm) MM cells. **C, D**. Confocal imaging and flow cytometry quantification of autophagy in U266 and MM1.S MM cells, compared to their KD^ULK3^ counterpart (U266 NTC, n = 10 vs. U266 KD^ULK3^, n = 16, unpaired t test, p = 0.04) and MM1.S NTC, n = 18 vs. MM1.S KD^ULK3^, n = 23, unpaired t test, p = 0.0002), measured by a cationic amphiphilic tracer (CAT) autophagic vacuoles dye (CYTO-ID^®^ Autophagy detection kit). Each value is the average/cell, measured over 3 different biological replicates of duplicated independent CRISPR knockout experiment. **E**. m-Cherry-GFP-LC3B expressing WM1366 melanoma cells were silenced for ULK3 and the impact on autophagy inhibition assessed by immunoblotting and confocal microscopy analysis. **F, G**. Red puncta indicate ongoing autophagy while yellow mcherry-GFP-LC3B colocalization indicates autophagy inhibition since GFP is not degraded. Each plotted value represents the median of 3 independent experiments (n = 5 fields/fluorodish, unpaired t test, p = 0.0267). Quantification was as follows: Normalized colocalization: (Sum Red and Green Overlap area (μm^2^)/cell count*100/Average red area per cell). Median is annotated; asterisks denote statistical significance. For immunoblots, numbers indicate molecular weight in kDa, and actin was used as a loading control. **H**. MM cell growth curves of parental vs. KD^ULK3^ counterparts (U266 NTC vs. U266 KD^ULK3^ and MM1.S NTC vs. MM1.S KD^ULK3^). Cell counts were determined by trypan blue dye exclusion over 12 days post transduction. Each dot represents the mean value of 2 biological replicates. U266 Two-way MIXED analysis adjusted p values: * p = 0.0138; ** p = 0.0024; ***p = 0.0003; ****p <0.0001). MM1.S Two-way MIXED analysis, adjusted p values: ** p = 0.054; ***p = 0.0004; ****p <0.0001).

To test if ULK3 is necessary for MM autophagy, we used CRISPR-CAS9 to ablate ULK3 in U266 and MM.1S cell lines and observed altered LC3-I/LC3-II ratios, indicating autophagy blockage (Fig. 2A, B). CYTO-ID staining also revealed significant reduction in functional autophagic vesicle formation in ULK3-knockdown cells (Fig. 2C, D). To confirm the role of ULK3 in autophagy, we employed adherent WM1366 cells bearing m-Cherry-LC3B/GFP-LC3B autophagy flux reporters. ULK3 knockdown was associated with impaired autophagy (Fig. 2E–G), resulting in accumulation of dysfunctional vesicles and cell death. Importantly, we also noted that the genetically induced reduction of ULK3 significantly compromised cell growth and survival over time (Fig. 2H). Overall, these data suggest a key role for ULK3 in MM autophagy.

### Development of novel small molecule kinase inhibitors targeting ULK3 and autophagy

Given the role of ULK3 in MM cell autophagy and the lack of currently available inhibitors, we developed new molecules to target its activity. To this end, we utilized kinase inhibitors developed at Moffitt that could inhibit ULK3 as well as other kinases and BET proteins like BRD4 [27, 28]. Compound SG3–014 (SG3; Supplementary Fig. 3A) inhibits BRD4, JAK2 and ULK3 with IC_50_ of 6.0, 4.1 and 162 nM, respectively (Supplementary Fig. 3A). Inhibition of ULK1 was considerably weaker (IC_50_ = 683 nM). Of note, BRD4 is highly expressed in MM [29–31], and we validated these observations in our dataset (Supplementary Fig. 3B). BRD4 is a known regulator of the MYC oncogene and can be inhibited by JQ1 [32]. However, resistance to JQ1 can develop quickly [33, 34]. We therefore posited that multi-kinase inhibitors targeting both BRD4 and ULK3 would prevent resistance. SG3 treatment led to rapid (6 hours) reductions of ULK3 and ULK1 (Supplementary Fig. 3C). This may involve proteasomal degradation or autophagic turnover. Of note, this was not observed with the BRD4 inhibitor JQ1 (Supplementary Fig. 3D). Moreover, SG3 treatment led to alterations of the LC3-I/LC3-II ratio (Supplementary Fig. 3E) and to marked reductions in autophagic flux and cell survival (Supplementary Fig. 3F–H). Given the impact of MM on bone destruction, we noted that SG3 could potently inhibit osteoclastogenesis while importantly having no effect on either mesenchymal stromal cells (MSCs), or osteoblasts (Supplementary Fig. 3I). Thus, we hypothesized these inhibitors could protect against cancer-induced bone disease.

**Figure 3.**
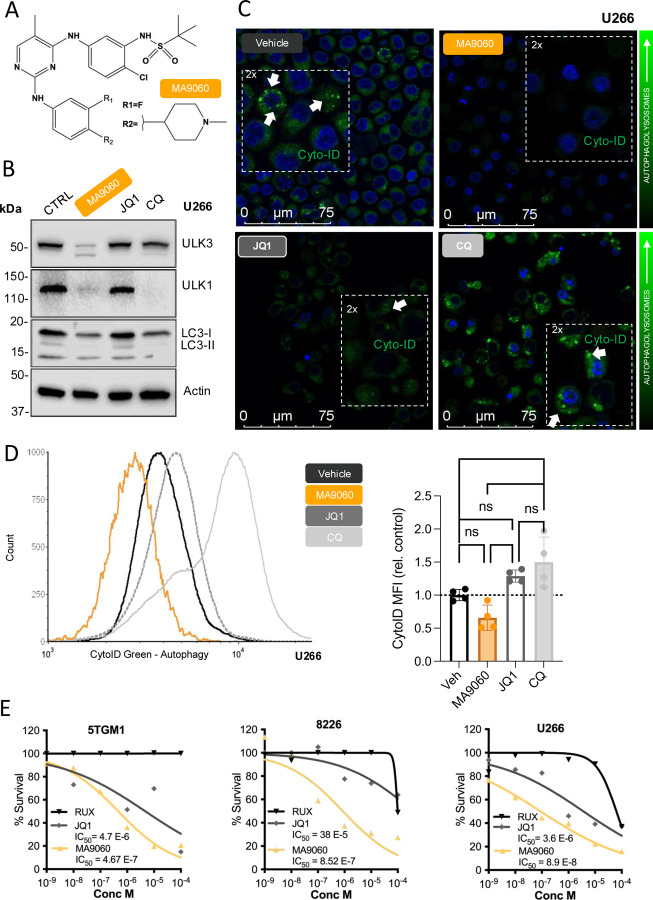
Anti-myeloma activity of the MA9–060 autophagy inhibitor. **A**. Chemical structure of the autophagy inhibitor MA9–060. **B**. Effects of MA9–060 (1μM) and JQ1 (10μM) (6h treatment) on the steady state levels of autophagy proteins in U266 MM cells. Chloroquine (CQ) (1μM) served as a positive control for autophagy inhibition. **C**. Representative confocal images (scale 1–75 μm) and flow cytometry quantification (**D**) of autophagy in U266 MM cells, treated with MA9–060, JQ1 or CQ measured by a cationic amphiphilic tracer (CAT) autophagic vacuoles dye (CYTO-ID^®^ Autophagy detection kit). Hoechst 33342 dye appears here as the nuclear blue staining (Multiple comparisons ANOVA, p <0.0001). **E**. MTS assay demonstrating the inhibitory profile of MA9–060 (10^−4^ to 10^−9^ M) on MM cells (5TGM1, 8226, U266).

In preparation for *in vivo* studies, *in vitro* hepatocyte microsomal stability assays revealed a short half-life for SG3 leading us to develop MA9–060 (MA9) (Fig 3A and Supplementary Fig. 4A, B) [35]. The molecular binding mode of MA9:ULK3 was determined by X-ray crystallography. The structure indicates that MA9 is a type-1 inhibitor engaging the hinge Cys94 residue via hydrogen bonding with its pyrimidyl N1 and 2-anilinyl NH group. The ULK3 DFG motif was found to be configured in a typical kinase inactive conformation (Supplementary Fig. 4C, D). Like SG3, MA9 treatment of U266 MM cells also provoked rapid reductions in ULK3 levels and autophagy effectors (Fig. 3B). Notably, U266 cells express little to no MYC but we validated the impact of MA9060 on ULK3 and MYC in the MM.1S cell line and showed MA9060 was not toxic to normal primary human MSCs in the nM concentration (Supplementary Figure 4E–H). We also demonstrated that MA9 treatment was comparable to CQ in significantly reducing autophagy (Fig. 3C and Supplementary Fig. 5) while JQ1 had no effect (Fig. 3C, D). Importantly, CQ inhibition of autophagic flux results in the accumulation of large vesicles while MA9 appeared to inhibit the proper formation of autophagolysosomes. Like SG3, MA9 treatment rapidly compromised cell survival *in vitro,* whereas ruxolitinib, a JAK2 inhibitor control, had no effect. These results suggest that the MA9 mechanism of action likely reflects autophagy blockade in the U266 model (Fig. 3E).

### MA9 significantly reduces MM growth and myeloma-associated bone disease *in vivo*

To assess the *in vivo* efficacy of MA9 and avoid MYC inhibition confounding our results, we employed the U266 MYC human cell line that reliably homes to the skeleton when tail vein injected into NSG mice [36]. Upon confirmation of engraftment by bioluminescence imaging (BLI), mice were randomized into treatment groups on Day 20, including a JQ1 treatment arm to assess the impact of BRD4 inhibition within the host microenvironment.

**Figure 4.**
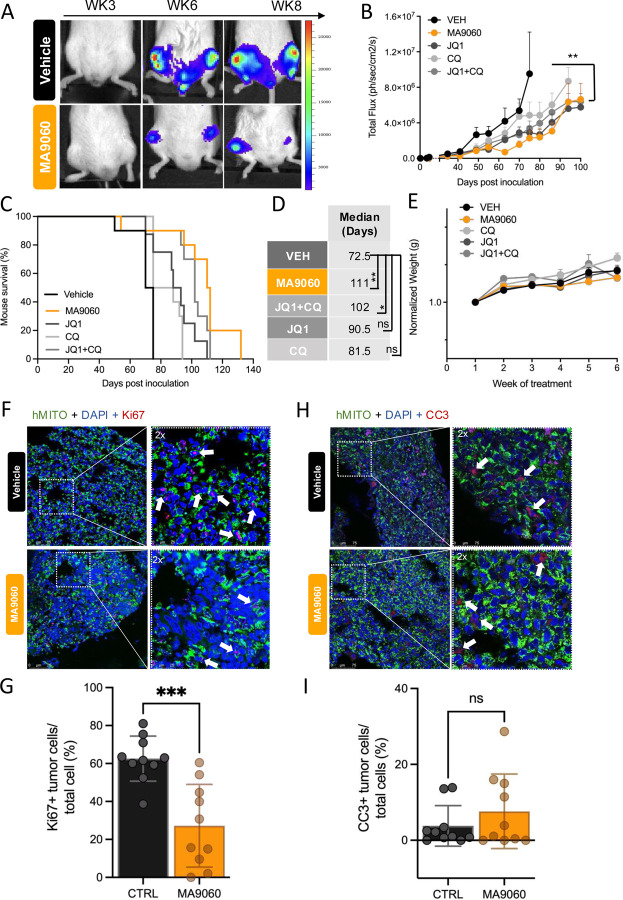
MA9–060 has potent in vivo anti-myeloma activity. **A**. Representative bioluminescent imaging (BLI) of the indicated cohorts of mice (n = 10/group) tail vein injected with 1 ×10^6^ U266-Luc MM cells and treated with vehicle control (**β**-HCD Captisol) or MA9–060. Treatment started at day 20 (M-F, 10 mg/kg/day subcutaneously). **B**. Bioluminescence quantification of tumor burden over 100 days post inoculation (VEH vs MA9060, Bonferroni’s adjusted p = 0.0073). **C**. MA9–060 treatment improves overall survival (median 111 days post-tumor inoculation) compared to vehicle control (CTRL, median 72.5 days). Of note, MA9–060 treatment did not show any sign of toxicity (weight loss <20%, ruffled hair) at the chosen dose. **D**. Table indicating median survival (days) and significance of VEH CTRL vs treatment groups (Log Rank Mantel Cox test, p < 0.0001). **E**. Weight monitoring (weekly) of the indicated cohorts. **F, G**. Representative confocal microscopy images (F) and quantification (G) of formalin fixed, paraffin embedded tumors (5-micron sections), stained for Ki67 and human MM cells (hMITO) colocalization. Alexa Fluor Plus 647 goat anti-rabbit and Alexa Fluor Plus 488 goat anti-mouse were used as secondary antibodies. Confocal immunofluorescent images were acquired with Leica TCS SP8 system with 63x objective (Leica) and quantified as number of Ki67+/hMITO+ cells, normalized to the total nuclei number of analyzed area (unpaired t test, p = 0.0003) **H, I**. Representative confocal microscopy images (H) and quantification (I) of formalin fixed, paraffin embedded tumors (5-micron sections), stained for CC3 and human MM cells (hMITO) colocalization. Alexa Fluor Plus 647 goat anti-rabbit and Alexa Fluor Plus 488 goat anti-mouse were used as secondary antibodies. (I) Confocal immunofluorescent images were acquired with Leica TCS SP8 system with 40x objective (Leica) and quantified as number of CC3+/hMITO+ cells, normalized to the total nuclei number of analyzed area (unpaired t test, p = 0.2915).

**Figure 5.**
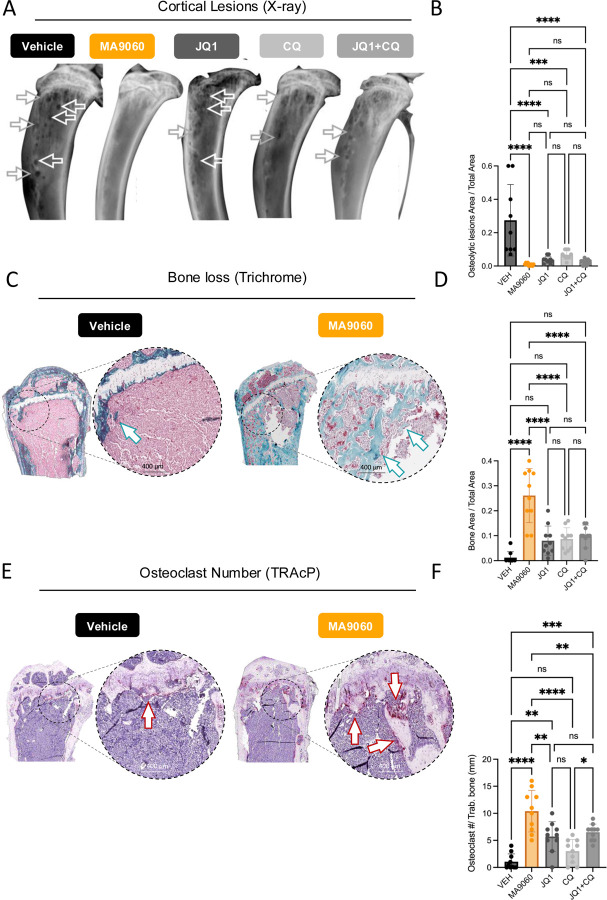
MA9060 treatment protects from bone-associated MM disease. *Ex vivo* quantification of MM associated bone destruction, measured in untreated mice (VEH) vs. treatment groups (MA9–060, JQ1, CQ, and JQ1+CQ cohorts). **A**. Representative cortical osteolytic lesions imaging and quantification (n = 10/treatment group), that were measured (**B**), by Faxitron x-rays (multiple comparisons ANOVA, p <0.0001). **C**. Histological analyses of bone sections (H&E staining, (n = 10/treatment group), and **D** relative quantification of trabecular bone (multiple comparisons ANOVA, p <0.0001). **E**. Histological analyses of osteoclast activity (TRAcP staining, n = 10/treatment group; indicated by arrows), and **F**, quantification of osteoclast number (multiple comparisons ANOVA, p <0.0001). Of note, the MA9–060 treated group shows higher actively resorbing osteoclast number on trabecular bone, that is not observed in the vehicle cohort, since the aggressive bone marrow MM destroyed the internal bone structure.

MA9 dosing was based on that of JQ1 [32, 37]. Our results demonstrate that MA9 treatment significantly impaired tumor growth compared to control (Fig. 4A, B). At endpoint, MA9 had nearly double the median survival (112 days) compared to vehicle (70 days) (Fig. 4C, D), outperforming other treatment arms, including JQ1+CQ (Fig. 4D). Importantly, there were no overt toxicities (weight loss, lethargy) with the applied dosing regimen (Fig. 4E). Consistent with *in vitro* results, MA9 significantly decreased ULK3 levels in *κ*λ light chain-expressing MM cells (Supplementary Fig. 6A, B). Moreover, MA9 reduced MM proliferation although no impact on apoptosis was observed at study endpoint when tumor burdens were similar in each group (Fig. 4F–I). Importantly, X-ray and histological analyses revealed that MA9 significantly protected against MM-induced osteolysis compared to other treatment arms (Fig. 5A–D). MA9 treated mice had higher numbers of osteoclasts per mm/bone compared to controls but this was primarily because little or no trabecular bone present in the control cohort (Fig. 5E, F). Thus, MA9 displays significant anti-myeloma activity and protects against cancer-associated bone disease.

To further demonstrate the importance of ULK3 in MM progression, we inoculated mice with wildtype and ULK3^−/−^ cells (Supplementary Fig. 7A, B). Our data show that loss of ULK3 resulted in a delayed onset of disease and significantly higher overall survival (Supplementary Fig. 7B, C). We also demonstrated the selectivity of MA9 for ULK3 (Supplementary Fig. 7D), showing that ULK3^−/−^ MM bearing mice received no further benefit in terms of overall survival (Supplementary Fig. 7E, F). These findings collectively highlight ULK3 as a functionally relevant therapeutic target in MM and support the efficacy and specificity of MA9 *in vivo*.

### Drug resistant MM cells express increased levels of ULK3 and are sensitive to MA9

**Figure 6.**
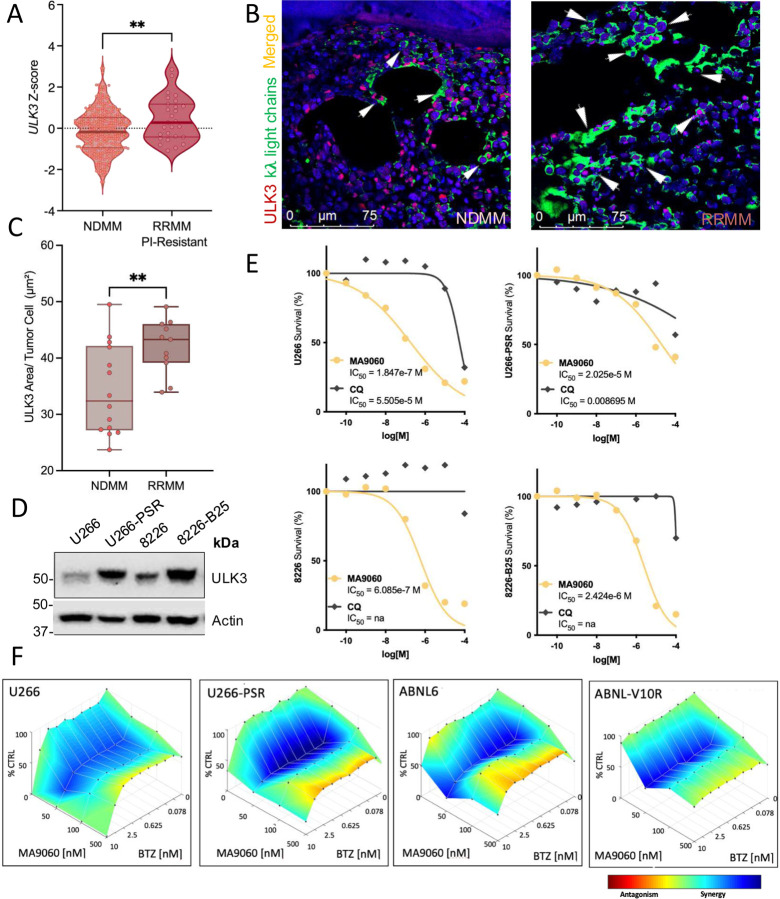
MA9–060 shows potency as a monotherapy and synergizes with proteasome inhibitors (BTZ) *in vitro*. **A.** *ULK3* expression in divided in NDMM (*n* = 182) *vs*. post-proteasome Inhibitor treatment (*n* = 27) in Moffitt PMRC MM Patient Dataset. Asterisks denote statistical significance (unpaired *t*-test, p = 0.005). **B**. Representative confocal microscopy images of a NDMM bone marrow biopsy core (*left*) and RRMM (*right*) from Moffitt TCGA program Tissue Micro Arrays (H&E stained, scale bar; 100 μm), (120 cores, 40 cases, 10 normal bone marrow tissue cores, 3 MM cell lines as CTRL, 0.6-mm biopsies) generated at the H. Lee Moffitt Cancer Center. kλ light chains^+^/ULK3+ immunofluorescence staining (kλ light chain in green and ULK3 in red), DAPI nuclear staining is in blue. Control IgGs were also used (Millipore Gt X Hu Ig k and λ CAT# AP-502, AP-506). Arrows represent colocalization of ULK3 protein and anti-human kλ light chain positive cells (scale bar; 0–75 μm). **C**. Quantitative analysis of TMAs (NDMM, *n* =14 and RRMM, *n* = 11). Asterisks denote significance (unpaired *t* test, p = 0.0098). **D**. ULK3 expression in proteasome inhibitor (bortezomib) sensitive (U266, 8226) *vs*. resistant (U266-PSR, 8226-B25) MM cell lines. **E**. MTS assays of the indicated MM cell lines following treatment with MA9–060 after 48h, expressed as IC_50_. CQ is here used as reference CTRL for autophagy flux inhibition. **F**. Proteasome inhibitor (Bortezomib, BTZ) sensitive (U266, ANBL6) and resistant counterpart (U266-PSR, ANBL6-V10R) MM cells were treated with BTZ (0–100nM) in combination with fixed doses of MA9–060 (50, 100, and 500 nM). Bluer colors indicate synergism at the defined doses. Synergy data is mapped as dose-response D-R (LOEWE) with Combenefit Software.

Proteasome inhibitors (PI) such as bortezomib (BTZ) and carfilzomib (CFZ) are standard treatments for MM, but resistance often develops [38]. Interestingly, analysis of Moffitt RNA-Seq data showed a significant increase in *ULK3* expression in MM patients who failed multiple lines of therapies, including PIs, compared to those that were newly diagnosed patients, (Fig. 6A). Tissue microarray (TMA) analysis confirmed elevated ULK3 protein in relapsed patients and PI-resistant MM cell lines (Fig. 6B–D) [39–41]. Moreover, *in vitro* cytotoxicity assays revealed that PI-resistant MM cells were highly sensitive to MA9 compared to CQ (Fig. 6E). We also noted that combination of MA9 with BTZ could significantly enhance MM toxicity in PI resistant cell lines which is in keeping with reports showing roles for autophagy in facilitating proteasome inhibitor resistance (Fig. 6F) [42–50].

### MA9 displays potent anti-myeloma activity against patient-derived MM cells *ex vivo*

**Figure 7.**
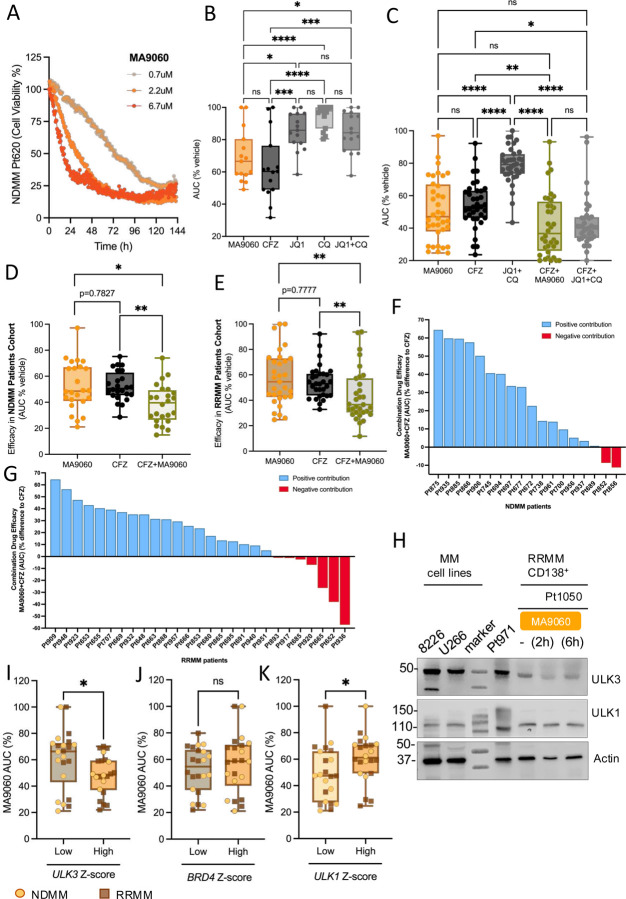
MA9–060 treatment compromises the survival of patient NDMM and RRMM cells *ex vivo*. **A**. Representative viability (live cell imaging) response of CD138^+^ cells from NDMM patient (Pt620) cultured and tested in autologous stromal microenvironment in EMMA’s ex vivo platform, exposed to three different concentrations of MA9–060 (0.7, 2.2, 6.7 μM). Of note, no toxicity was observed for co-cultured stromal cells, even at the highest concentration of MA9–060. (not shown here – 20 μM). **B**. *Ex vivo* EMMA platform assay to quantify the chemosensitivity of primary CD138^+^ MM cells plated in a collagen matrix with bone marrow stroma and patient plasma. Tumor cells were treated with carfilzomib (CFZ), MA9060, JQ1, CQ and JQ1+CQ and efficacy (live cell imaging) was measured as Area Under the Curve (AUC % to vehicle control) for 96 hours (EVOS FL Auto) (Tukey’s multiple comparison test, *n* =15 patients). **C**. The duplet combination therapy of MA9–060 and CFZ was tested *vs*. the triplet JQ1+CQ+CFZ combination, showing equal efficacy in MM patients (Tukey’s multiple comparison test, *n* = 36). **D, E**. Combination therapy of MA9–060 and CFZ is highly efficacious in paired analysis of NDMM (n = 18) (**D**) and RRMM (*n* = 27) (**E**) patients (Tukey’s multiple comparison test). **F, G**. NDMM (**F**) and RRMM (**G**) are represented as single patient response to combination of MA-9060 + CFZ. The contribution effect of MA9060+CFZ treatment over CFZ single agent independent treatment (0) is quantified and displayed as positive (blue bars) (NDMM, n = 18; RRMM, *n* = 20) or negative (red bars) (NDMM, *n* = 2; RRMM, *n* = 7), AUC % difference MA9060+CFZ *vs*. CFZ. **H**. Immunoblot showing levels of indicated proteins in CD138^+^ cells isolated from RRMM patients (Pt971, Pt1050). Pt1050 CD138^+^ cells were assessed for ULK3-mediated autophagy related proteins (ATGs), following treatment with MA9–060 (1μM at 2 and 6 hours). **I**. RNA-seq data of Moffitt MM patients (*n* = 44, 19 NDMM, 25 RRMM) divided in *ULK3*^high^ and *ULK3*^low^ (≤ median) expression (Z-score) and correlation to MA9–060 treatment response (AUC) (unpaired *t* test, p = 0.0042; Wilcoxon Test p = 0.0182). **L**. RNA-seq data of Moffitt MM patients (*n* = 44, 19 NDMM, 25 RRMM) divided in *BRD4*^high^ and *BRD4*^low^ (≤ median) expression (Z-score) and correlation to MA9–060 treatment response (AUC) (unpaired *t*-test p=0.4331).

For clinical robustness, we tested the activity of MA9 in MM patient samples using our *ex vivo* mathematical myeloma advisor (EMMA) platform (Supplementary Table 1) [6, 23, 51, 52]. EMMA allows drug testing as single therapies or in combination, on patient derived CD138^+^ selected MM cells cultured with collagen I and patient-derived bone marrow stroma over 6 days using live cell imaging. An area under the curve (AUC) up to 96 hours for each dose is calculated and results are displayed as a mean efficacy AUC. Using this approach, we noted that MA9 treatment potently induced the death of patient derived MM cells (Fig. 7A). Parallel samples from 15 MM patients exposed to different chemotherapeutics showed that MA9 is a potent single agent, even outperforming the duplet JQ1+CQ combination. Further, MA9 showed comparable potency versus PI (CFZ) across the same MM patient specimens (Fig. 7B). These findings were independently validated in a second cohort of MM patients (*n* = 36), demonstrating that both MA9 and CFZ treatment were similarly potent in compromising MM cell survival, and that the combination of MA9 with CFZ deepened the cytotoxic response, in a manner comparable to the triplet treatment of JQ1+CQ+CFZ (Fig. 7C, Supplementary Table 2–3). Moreover, comparing the MA9 response in NDMM (*n* = 18) and RRMM (*n* = 27) patients revealed that MA9 was effective as single agent but, when combined with CFZ, significantly improved cytotoxicity in both groups (Fig. 7D, E). A waterfall plot showed CFZ combined with MA9 yielded a positive contribution to efficacy of CFZ alone in 88% of NDMM and 74% of RRMM patients (Fig. 7F, G). We also demonstrated MA9 potency in provoking the rapid suppression of ULK3 within a single patient specimen (Fig. 7H).

To determine whether a correlation between *ULK3* expression and MA9 efficacy existed, we analyzed paired RNA-Seq data for each sample undergoing EMMA analysis and observed that patients with ULK3 levels higher than the median value (*n* = 44, 19 NDMM, 25 RRMM) had a significantly better response to MA9, suggesting that the efficacy of MA9 is in part due to *ULK3* inhibition (Fig. 7I, Supplementary Table 4). This was supported by data showing that MA9 efficacy did not correlate with *BRD4* expression levels (Fig. 7J, Supplementary Table 4). Patients with low *ULK1* expression had a significantly better response to MA9 than those with higher *ULK1* levels (Fig. 7K, Supplementary Table 4). Taken together, these data suggest that targeting ULK3 could be a highly effective treatment strategy for newly diagnosed MM or MM patients that have relapsed on PI treatment.

## DISCUSSION

Our findings establish ULK3 as a critical kinase that can initiate autophagy in MM. Furthermore, ULK3 plays a fundamental role in supporting MM cell survival and disease progression, highlighting its potential as therapeutic target. Using the small molecule inhibitor MA9, we demonstrate that ULK3 activity can be effectively blocked at nanomolar concentrations, leading to a reduction in MM tumor burden, preservation of bone integrity, and improved overall survival *in vivo*. Importantly, we demonstrate the robustness of our findings in isolated patient CD138^+^ MM cells *ex vivo*, further reinforcing the clinical relevance of ULK3 as a therapeutic target.

The specific substrates of ULK3 and the mechanism by which it promotes autophagy remains to be elucidated. Interestingly, ULK3 protein levels decline sharply within 6 hours of inhibitor treatment, suggesting this kinase may be subject to proteasomal degradation. The ubiquitin proteasome system (UPS) and autophagy programs are tightly linked as cell survival mechanisms, and we suspect that inhibition of ULK3 by MA9 results in proteasomal degradation, but this is yet to be validated [53]. While both ULK1 and ULK3 participate in autophagy initiation, the lack of functional compensation by ULK1 following ULK3 loss suggests these kinases respond to distinct cellular cues or stresses [54]. It should be noted that ULK3 has also been implicated in other important aspects of cellular biology such as acting as a quality control for cellular abscission and cell cycle progression [16, 55–58]. Other reports have linked ULK3 to the expression of oncogenes such as GLI2 [59], which induces the expression of anti-apoptotic proteins, again tying in with the role of autophagy in cancer cell survival [60].

Therapeutically targeting autophagy in cancer has proven difficult, in part due to non-specific drugs like chloroquine, which have off-target effects and poor tolerability. Clinical trial results with CQ thus far have been underwhelming, but this may be because the mechanisms of action of CQ are still not fully understood with toxicity making the treatment difficult for patients to tolerate long term [61, 62]. Therefore, more precise pharmacologic targeting of key nodes that control autophagy may result in better translation to the clinic. In this regard, we believe ULK3 is an ideal pharmacologic target not only because it appears to be induced under chemical stress as opposed to nutrient starvation [54], but also, along with ATG13 and FIP200, our proteomics data identify ULK3 as a key initiator of autophagy.

Genetic silencing of ULK3 significantly reduces autophagy in MM despite changes in ULK1 expression, again underscoring a lack of functional redundancy amongst ULK family members. Multi-kinase inhibitors used herein were originally intended to block both JAK2 and BRD4. Using the JAK2 inhibitor ruxolitinib as a starting point, SG3 and MA9 were found to retain their JAK2 inhibition profiles [27]. However, the MM cell lines tested here were not affected by the JAK2 inhibitor ruxolitinib, indicating the primary mechanism of action of SG3 and MA9 in compromising MM cell survival via inhibition of ULK1/3 or BRD4. JQ1 inhibition of BRD4 did not impact autophagy in the variety of assays performed.

*In vivo* analysis with the U266 cell line, characterized by negligible expression of the MYC oncogene, showed MA9 outperformed JQ1 by extending median overall survival, suggesting that ULK3/ULK1 blockade is responsible for improved efficacy. Notably, MA9 showed similar performance to the JQ1 plus CQ combination *in vivo*, but MA9 has the advantage of being administered as a single agent thus avoiding potential pharmacokinetic/pharmacodynamic issues involved in the administration of multiple reagents. Of note, while we did observe reduced MM proliferation in the MA9 treatment arm, we only observed a trend towards increased apoptosis. We posit that this may be due to clonal selection *in vivo* and we would expect that removing the mice from the study at earlier time points would reveal more apoptosis in the MA9 group compared to vehicle control. A caveat of our MA9 studies is it also inhibits ULK1, albeit it at a higher IC_50_ than ULK3 (253nM for ULK3 *vs*. 1.71μM for ULK1). Thus, we cannot rule out that some of the observed *in vivo* effects are due to ULK1 inhibition. The kinase domain of ULK3 crystal structure has recently been published and should allow for the continued development of ULK3 specific inhibitors [63]. *In vivo* studies with ULK3 ablated MM cells showed delayed disease progression and prolonged survival, demonstrating ULK3’s role in MM, while resistance to MA9 in ULK3 KO cells confirmed the inhibitor’s on-target activity and therapeutic potential.

Finally, MM patients typically are given multiple chemotherapies often in combination with proteasome inhibitors [4]. This was true for the MM patient specimens studied herein, where CD138^+^ patient cells used for *ex vivo* assays had been exposed to multiple treatments. Regardless of their history, we observed significant responses to MA9 across the disease spectrum of MM, indicating that MA9 may be able to overcome multiple mechanisms of resistance, which requires further investigation. To date, we have not been able to assess MA9 in patients who have failed immunotherapies such as CAR-T. However, emerging evidence coming from bulk RNA sequencing of clinical samples suggests that increased autophagy promotes resistance to CAR-T [64]. With the crystal structure of the ULK3 kinase domain having been resolved, our studies should initiate the generation of selective ULK3 inhibitors that will prove highly beneficial for the treatment of MM patients across the disease spectrum.

In summary, our data highlight ULK3 as a key mediator of MM autophagy regulation and cell survival. Targeting ULK3 represents a promising therapeutic strategy, particularly in resistant disease settings, and provides a foundation for future drug development efforts focused on autophagy-dependent cancers.

## MATERIALS AND METHODS

### Study design

To uncover the role of ULK3 mediated autophagy in multiple myeloma we used primary cells, TMA samples from patients and healthy donors, and preclinical validation in mice and cell lines. Ethical considerations are described in the Supplementary Materials.

### Study Participants

Peripheral blood and bone marrow (BM) samples were obtained from IRB consented patients (MCC14690 and MCC18608). Specimens were handled per the Declaration of Helsinki, the International Ethical Guidelines for Biomedical Research Involving Human Subjects (CIOMS), the Belmont Report, and the U.S. Common Rule. See Supplementary Table 1 – Patient Demographics for patient demographics.

### CD138^+^ Patient cell isolation and purity

Bone marrow (BM) aspirates (20ml) were collected and bone marrow mononuclear cells (BMMCs) isolated using standard procedures. BMMCs were enriched for CD138 using Miltenyi (Bergisch Gladbach, Germany 130-051-301) antibody-coated magnetic beads and cytospin slides of the CD138+ cells were prepared. Post-isolation, the average purity was 95.3% (IC: 88.1% - 100%).

### RNA Sequencing (RNA-Seq)

After CD138^+^ cell enrichment, vials containing 1.0 × 10^6 viable frozen CD138^+^ cells were sent for RNA-Seq analysis. Additional details are included in Supplementary Materials.

### Data Mining

We extrapolated gene expression data from MM patients: MGUS, *n* = 64; SMM, *n* = 57; NDMM, *n* = 207; ERMM, *n* = 303; LRMM, *n* = 182 patient cohorts. We used the NCBI Gene Expression Omnibus (GEO; https://www.ncbi.nlm.nih.gov/geo/) to validate data for *ULK1*, *ULK2*, *ULK3*, *ULK4* mRNA expression from FPKM genome wide analysis; GSE5900 combined with GSE2658 (MGUS, *n* = 44; SMM, *n* = 12; NDMM, *n* = 559 patient cohorts, accessed in July 2023) [25, 65, 66].

### CD19^+^ B cell isolation

Human normal CD19^+^ B cells were isolated from healthy donors' peripheral blood mononuclear cells (IRB Pro00021733) using CD19-specific microbeads and LS columns (Miltenyi Biotech, CAT# 130-050-301, CAT# 130-042-401) following the manufacturer's protocol.

### Immunoprecipitation & Immunoblotting

In co-immunoprecipitation studies, 5×10^6^ U266 cells (2 mg total protein) underwent lysis in RIPA buffer containing protease inhibitors. Samples were precleared with Agarose G protein beads (10 μl, CAT# 15920–010) and incubated for 1-hour at 4°C with 1 μg of ATG13 antibody (CAT# MBS8564879). After overnight incubation with beads, the pellet was collected, washed, and split for immunoblotting and proteomics analysis. Details on the LC-MS/MS analysis and immunoblotting can be found in the supplemental materials. Further, densitometry values for western blots can be found in Supplementary Figures 10 and 11.

### Immunofluorescence staining

Immunofluorescent (IF) detection of ULK3 was performed in MM patient tissue microarrays, fixed *in vitro* cell lines and *ex vivo* tissues derived from animal studies. We conducted IF staining for Ki67 and cleaved caspase-3 in *ex vivo* tissue sections derived from animal studies. Further details are provided in the Supplementary Materials.

### Reagents

Dual inhibitors SG3–014 and MA9–060, along with BRD4 inhibitor JQ1, were synthesized and characterized at the H Lee Moffitt Cancer Center, *US patent issued 2018; US10,106,507B2*. Chloroquine (CQ, CAT# C6628–25G) and Temsirolimus (Tems, CAT# PZ0020–5mg, ≥98% HPLC) were purchased from Millipore (Sigma-Aldrich). Ruxolitinib was purchased from Selleckchem (CAT# S1378). For *in vivo* use, all compounds were dissolved in PBS solution of 15% 2-hydroxypropyl-β-cyclodextrin (HPβCD; CAT# 128446-35-5, Millipore Sigma). Proteasome inhibitors, bortezomib (PS-341 CAT# 179324-69-7) and carfilzomib (PR-171 CAT# 868540–17-4) were purchased from Selleckchem.

### Viability, proliferation, and dose response assays

Myeloma cell lines were plated in 96-well plates (2 × 10^5^ cells/ml) and treated with vehicle control (HPβCD) and test compounds. Cell viability was assessed at 48 hours using the MTS assay (CellTiter 96, CAT# G3582). Absorbance was measured at 490 nm after a 2-hour incubation at 37°C. IC_50_ values were calculated using Prism software (version 9.3.1), and synergy matrices were generated with Combenefit Software [88].

### Genetic manipulation of ULK3 expression

We used standard CRISPR-CAS9 and siRNA approaches to knock down the expression of ULK3 in MM cell lines. The detailed protocols are included in Supplementary Materials.

### Measurement of autophagic activity - CytoID Assay

Autophagic activity was monitored using the Cyto-ID Green Autophagy Detection Kit (Enzo Life Sciences, ENZ-51031-K200, USA) as per manufacturer’s instructions. Further details and protocols for quantitative analysis by Flow Cytometry and Confocal Microscopy are provided in Supplementary Materials.

### *In vivo* preclinical multiple myeloma murine models

For the U266-Luc model, 1 × 10^6^ cells (100 μl/PBS) were tail vein injected into 4- to 6-week-old female and male NSG mice. Upon engraftment, mice were randomized into vehicle (*n* = 6), MA9–060, JQ1 (*n* = 9, 10 mg/kg, 5x/week, Mon-Fri), CQ (*n* = 10, 10 mg/kg, 5x/week, Mon-Fri), and JQ1+CQ (*n* = 9, 10 mg/kg JQ1 +10 mg/kg CQ, 5x/week, Mon-Fri) groups. Endpoint was represented by hind limb paralysis or >20% weight loss. When sacrificed, tumors tibiae were harvested and fixed in 10% neutral buffered formalin for 24 hours. Bioluminescent signal is reported as the average of the sum of dorsal and ventral signal of the mice/group/timepoint.

### *Ex vivo* drug sensitivity characterization and synergy studies

For these assays, we used a novel EMMA platform developed at Moffitt [51]. Further details are included in the supplemental Materials.

### Statistical analysis

Statistical significance in this study was assessed using MATLAB, Prism (GraphPad software), and IBM Statistical Package for the Social Sciences (SPSS) software. Graphs were generated using Prism (GraphPad) version 9. All sequencing data underwent analysis using R version 4.1.2 and Python version 3.10.1. Further details are provided in the Supplementary Materials.

## Supplementary Material

1

## Data Availability

The authors confirm that the data supporting the findings of this study are available within the article and its Supplementary Materials. ULK3-MA9060 crystal parameters, data collection statistics and refinement statistics are summarized in Supplementary Fig. 4D and deposited in the protein databank with PDB code 8V1M. The RNA-seq data used in this article is shared on Synapse (RRID:SCR_006307), a data sharing platform operated by Sage Bionetworks founded on FAIR (findable, accessible, interoperable, reusable) principles, and provides HIPAA-compliant security and data governance tools to ensure research data is protected and accessible to scientists while respecting patients’ rights. The log2FPKM and z-normalized RNA-seq data is publicly available for download at https://doi.org/10.7303/syn52316987. The molecular data used in this research was generated through private funding by Aster Insights (www.asterinsights.com) in collaboration with the Oncology Research Information Exchange Network (ORIEN, www.oriencancer.org). Requests for access to the raw data (FASTQ/BAM) used in this study can be submitted here at https://researchdatarequest.orienavatar.com/. RNASeq and disease sample mapping is available in Supplementary Table 6.

## Statement of significance:

This work uncovers a previously unrecognized role for ULK3 in driving multiple myeloma progression and treatment resistance. By linking ULK3 expression to advanced disease stages and demonstrating its functional role in autophagy and cell survival, we highlight ULK3 as a viable therapeutic target. Using newly developed inhibitors, we show that blocking ULK3 not only slows tumor growth and protects bone *in vivo* but also restores sensitivity to proteasome inhibitors in resistant myeloma cells. These findings provide a strong rationale for ULK3 inhibition as a new treatment approach in both early and relapsed/refractory multiple myeloma.
