# Supplementary material for: Unc-51 Like Kinase 3 (ULK3) is essential for autophagy and cell survival in multiple myeloma
Source: Res Sq. 2025 Aug 12:rs.3.rs-7160521. Preprint. [Version 1] doi: 10.21203/rs.3.rs-7160521/v1 (PMC12363935; doi:10.21203/rs.3.rs-7160521/v1)
Supplement: 1 [file NIHPPRS7160521V1-supplement-1.pdf]

SUPPLEMENTARY FIGURES AND TABLES

ULK3 is essential for autophagy and cell survival in multiple myeloma

Marilena Tauro[1], Tao Li[1], Praneeth R. Sudalagunta[2], Mark Meads[1,4], Rafael Renatino Canevarolo[2], Niveditha Nerlakanti[1] Raghunandan R. Alugubelli[4], Harshani R. Lawrence[6], Steven Gunawan[5], Mohammad Ayaz[5], Pradeep Nareddy[5], Sang Young Yun5, Gemma Shay[1], Kathy Yang[6], Timothy H. Tran[6], Ryan T. Bishop[1], Mostafa M. Nasr[1,3], Nicholas N.J. Lawrence[5], Ernst Schonbrunn[5], John L. Cleveland1, Ariosto S. Silva[3], Kenneth H. Shain[1,4] and Conor C. Lynch[1]*

[1]Department of Tumor Microenvironment & Metastasis, H. Lee Moffitt Cancer Center and Research Institute, Tampa, FL, USA.
[2]Department of Metabolism and Physiology, H. Lee Moffitt Cancer Center and Research Institute, Tampa, FL, USA.
[3]Cancer Biology Ph.D. Program, University of South Florida, Tampa, FL, USA
[4]Department of Malignant Hematology, H. Lee Moffitt Cancer Center & Research Institute, Tampa, FL, USA.
[5]Drug Discovery Program, H. Lee Moffitt Cancer Center and Research Institute, Tampa, FL, USA
[6]Chemical Biology Core, H. Lee Moffitt Cancer Center and Research Institute, Tampa, FL, USA

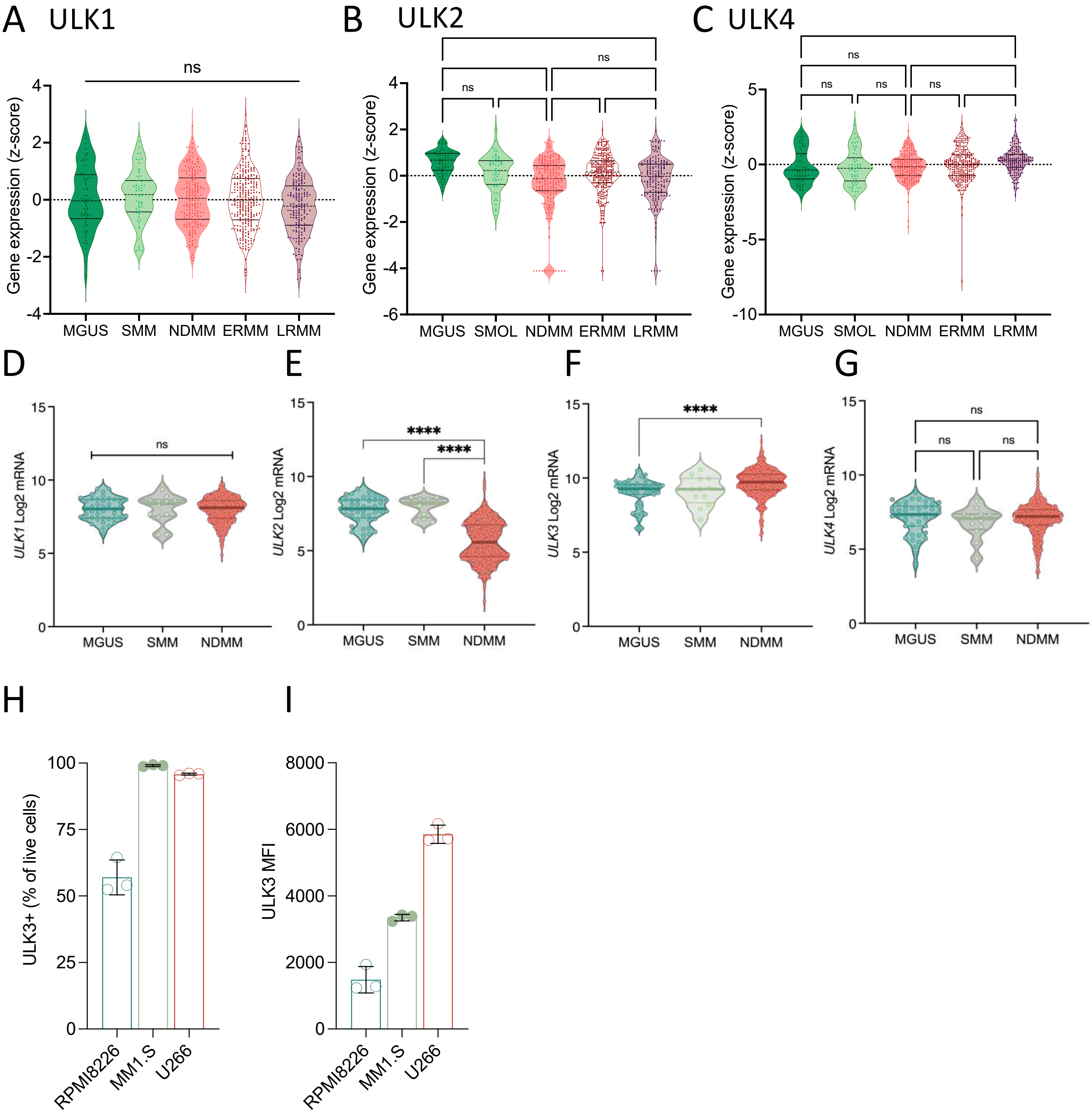

Supplementary Figures - Figure 1

Supplementary Figure 1. Increased *ULK3* gene expression levels correlate with MM progression stage. A-C. *ULK1*, *ULK2*, *ULK4* isoforms expression (Z-score) of Moffitt MM patients divided into disease stage cohorts, Monoclonal Gammopathy of Undetermined Significance (MGUS; *n* = 64), Smoldering MM (SMOL; *n* = 57), Newly Diagnosed (NDMM; *n* = 207), Early Relapsed (ERMM; *n* = 303) and Late Relapsed MM (LRMM; *n* = 182). Asterisks denote significance (Significance: (****) P<0.0001, (***) P<0.001, (**) P<0.01, (*) P<0.05, One-way ANOVA followed by Tukey's test). **D-G**. *ULK1*, *ULK2*, *ULK3*, and *ULK4* mRNA FPKM genome wide analysis from GSE5900 (MGUS *n* = 44; SMM *n* = 12)) + GSE2658 (NDMM *n* = 559) patient cohorts. Asterisks denote significance (Kruskal-Wallis, *ULK1*, p = 0.6068; *ULK2*, p <0.0001; *ULK3*, p <0.0001; *ULK4*, p <0.0001). **H-I** Flow cytometry quantification of ULK3 expression across different MM cell lines: 8226 (RPM8226), MM1.S, and U266.

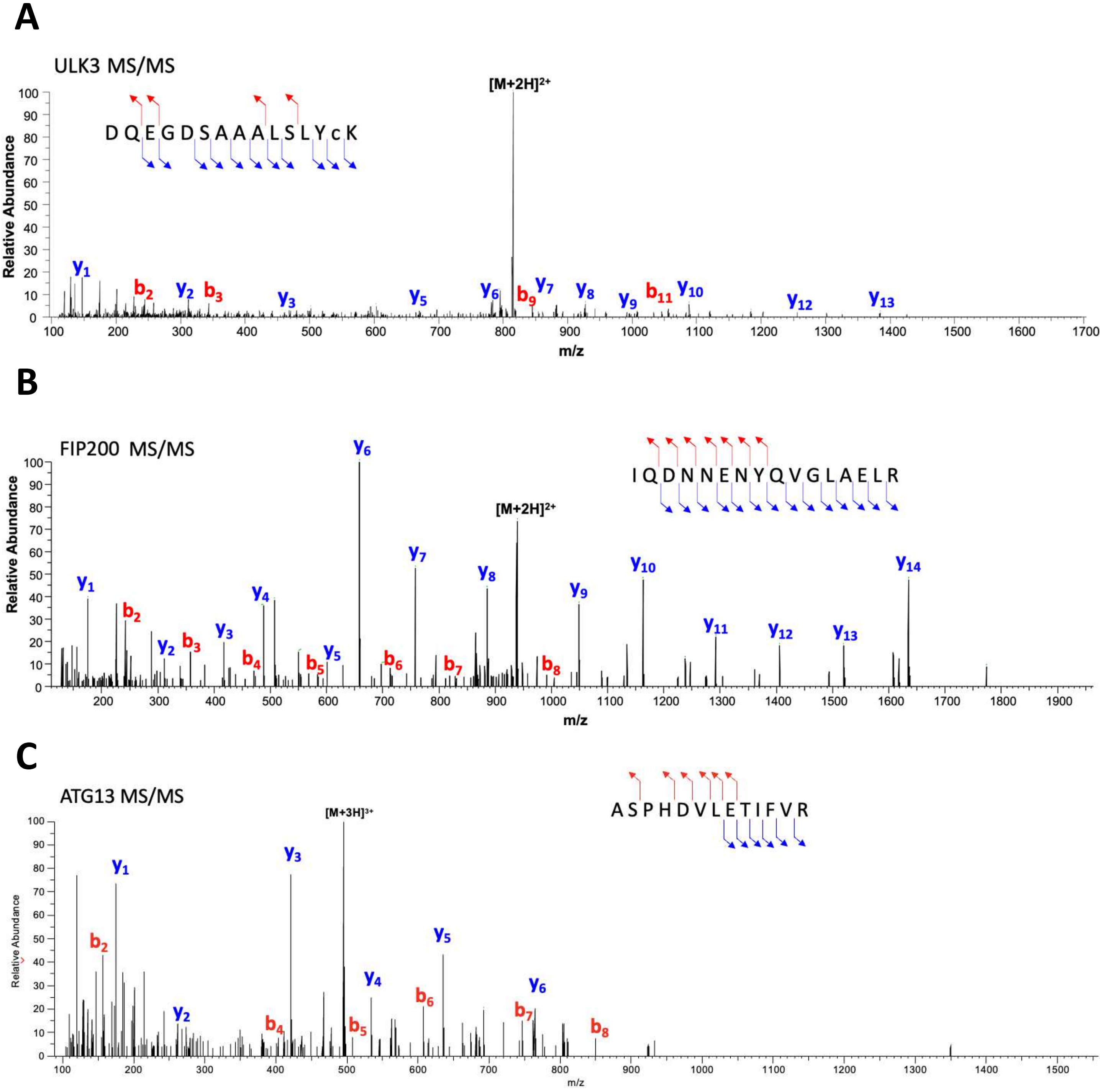

Supplementary Figures - Figure 2

Supplementary Figure 2. ATG13 immunoprecipitation to Mass Spectrometry Targeted Proteomic identification of ULK3 protein complex in U266 cells.

A. LC-MS/MS mass spectrometry identification of ULK3. ULK3_DQEGDSAAALSLYcK was set as PRM target (814.37 m/z, charge state 2+); this result was also confirmed with protein database searching with Proteome Discoverer 3.0, with a Sequest XCorr score 2.70, and a precursor mass measurement accuracy of -0.096 ppm. **B.** LC-MS/MS identification of FIP200. FIP200_ IQDNNENYQVGLAELR was set as PRM target (938.46 m/z, charge state 2+); this result was also confirmed with protein database searching with Proteome Discoverer 3.0, with Sequest XCorr score 2.80, precursor mass measurement accuracy of -0.088 ppm. **C**. LC-PRM identifies ATG13. ATG13_ASPHDVLETIFVR was set as PRM target (495.27 m/z, charge state 3+), the result was also confirmed with Protein database searching with Proteome Discoverer 3.0, PD XCorr score 3.44, precursor mass measurement accuracy of 0.016ppm.

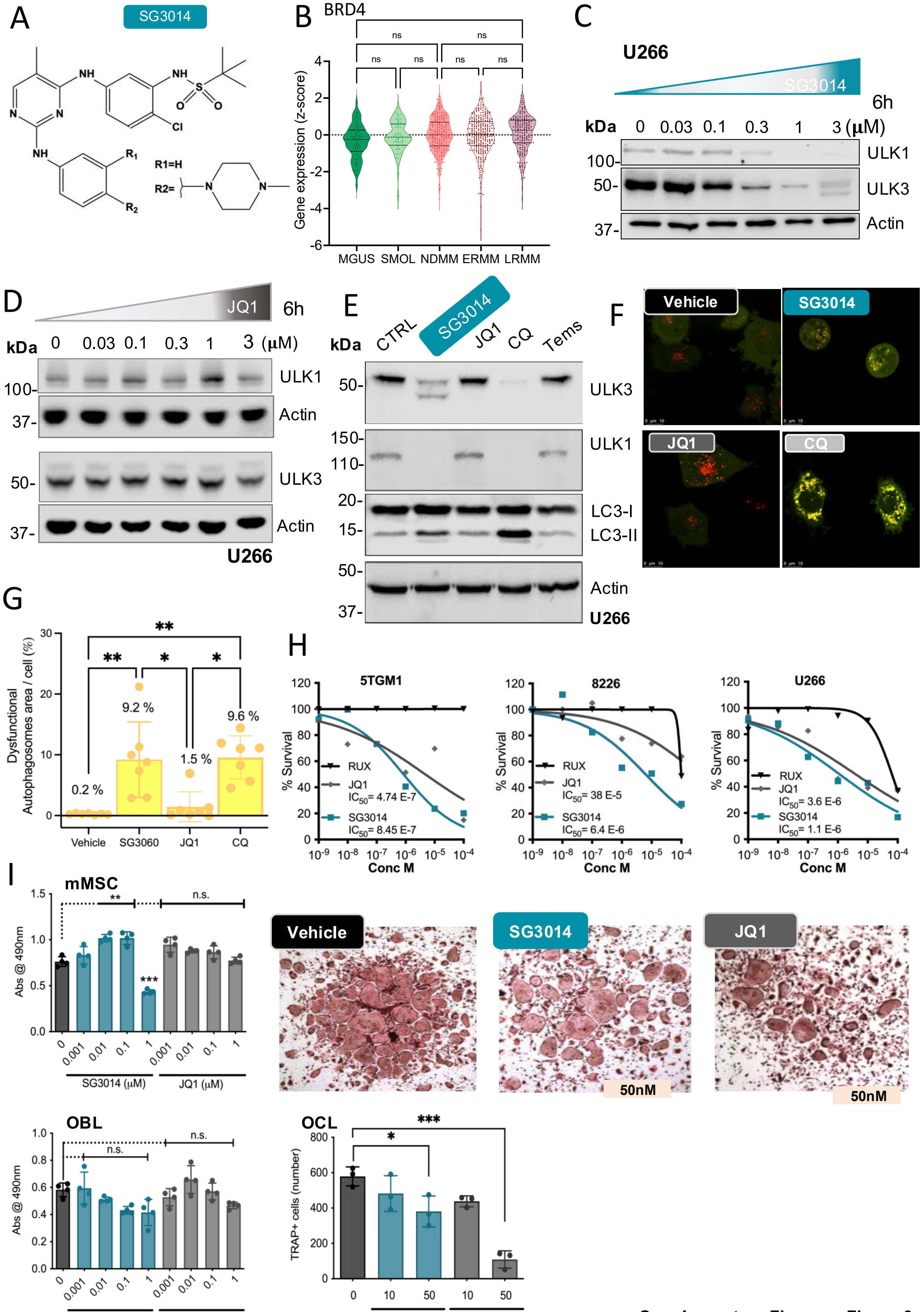

Supplementary Figures - Figure 3

Supplementary Figure 3. **Characterization of the novel autophagy inhibitor SG3-014. A**. Chemical structure of autophagy inhibitor SG3-014. **B**. *BRD4* gene expression in Moffitt MM Patient Dataset, with MM patients divided in disease stage cohorts. Data are represented as violin plots. Asterisks denote significance (Significance: (****) P<0.0001, (***) P<0.001, (**) P<0.01, (*) P<0.05, One-way ANOVA followed by Tukey's test). **C**. SG3-014 treatment (0-3 μM) decreases ULK3 protein levels in U266 MM cells (6h). JQ1 is here included as a control for BRD4 regulation (**D**). **E**. The effects of SG3-014 (1μM) and JQ1 (10μM) (6-hour treatment) on steady state levels of the indicated autophagy proteins in U266 MM cells. Chloroquine (CQ) (1 μM) served as a positive control for autophagy inhibition. Temsirolimus (Tems) (1 μM), an mTOR inhibitor, is included as a control for autophagy induction. **F.-G**. The effects of SG3-014 treatment on autophagic flux. m-Cherry-GFP-LC3B expressing WM1366 melanoma cells were treated with SG3-014 novel inhibitor and effects on autophagic flux were quantified by confocal microscopy analysis (*n* = 7). Red puncta indicate ongoing autophagy while yellow mcherry-GFP-LC3B colocalization indicates autophagy inhibition (dysfunctional autophagosome formation) since GFP is not quenched in the acidic autophagosome pH. Quantification (**G**) is expressed as normalized colocalization: (sum of red and green overlap area (μm2)/cell count*100/average red area per cell). Median is annotated. JQ1 were CQ used as controls. (Multiple comparisons ANOVA test, p <0.0001). **H**. MTS assay showing the inhibitory profile of SG3-014 on MM cells (5TGM1, 8226, and U266). Of note, MM malignancy is not dependent on JAK2 in the models used, as Ruxolitinib treatment shows no effect in this assay. **I**. MTS assay showing effects of SG3-014 treatment (0.001-1 μM) on mesenchymal stem cells (MSCs) and MC3T3-osteoblasts (OBL). JQ1 is used here as a control. Representative images of primary osteoclasts (OCLs; red; TRAcP staining positive) cultured in the presence of SG3-014 (50 nM) are also shown. JQ1 is used here as a control. Quantification is shown in histograms (*n* = 3/treatment condition, multiple comparisons ANOVA test, p <0.0001).

A

IC_{50} (nM)	CQ	JQ1	SG3014	MA9060
ULK1	NA	NA	683	1710
ULK2	NA	NA	858	4300
ULK3	NA	NA	162	253
BRD4	NA	7.2	6.0	3.8
JAK2	NA	NA	4.1	6.7

B

Microsomal Stability (%)

	Human	Mouse
SG3014	53.9	3.5
MA9060	43	100

C

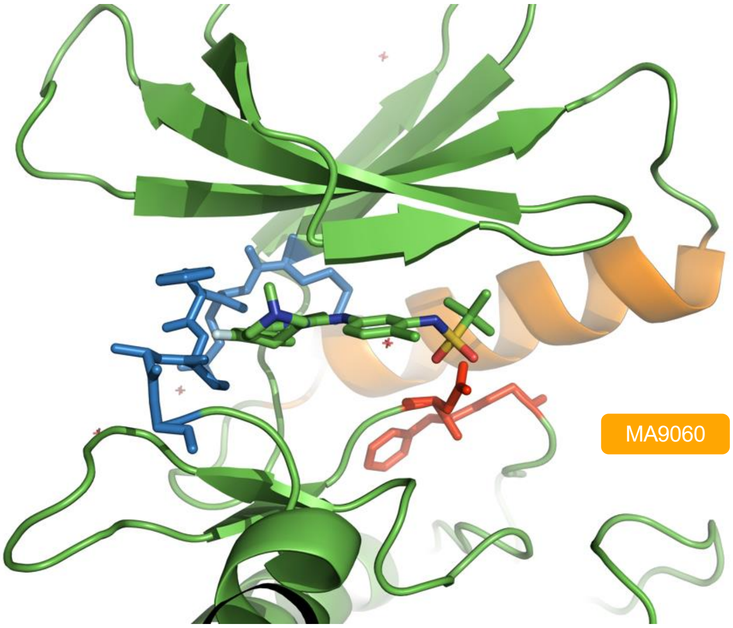

D

Data collection	hsULK3- MA9060
Resolution range (Å)	24.52 - 2.79 (2.89 - 2.79)
Space group	P62
Unit cell<br>a, b, c (Å)<br>α, β, γ (°)	84.73 84.73 78.03<br>90 90 120
Total reflections	59869 (4579)
Unique reflections	8014 (804)
Multiplicity	7.47 (5.70)
Completeness (%)	99.9 (100.0)
Mean I/sigma(I)	11.21 (1.64)
Wilson B-factor (Å^2)	76.16
R-merge	0.141 (2.235)
CC1/2	0.995 (0.346)
**Refinement**	
Reflections used	7998(804)
Reflections used for R-free	400 (40)
R-work	0.2049 (0.3532)
R-free	0.2773 (0.4376)
Number of non-H atoms	2125
macromolecules	2082
ligands	38
Protein residues	267
RMS - bond length (Å)	0.010
RMS - bond angle (°)	1.24
Ramachandran statistics favoured (%)	94.34
allowed (%)	5.66
outliers (%)	0.00
Average B-factor (Å^2)	84.93
macromolecules	85.17
ligands	70.59

E

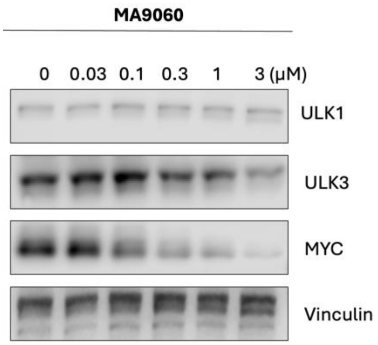

F

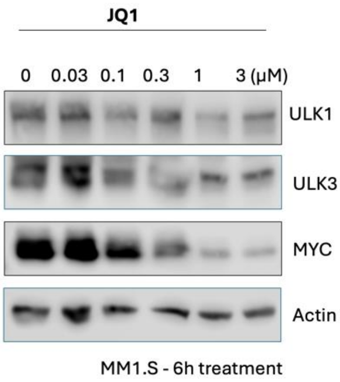

G

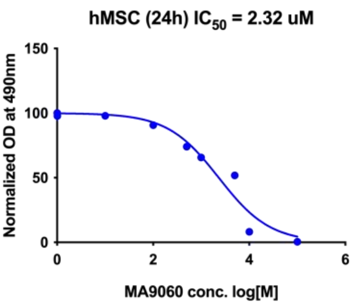

H

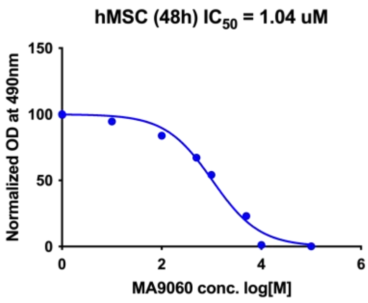

Supplementary Figures - Figure 4

Supplementary Figure 4. Pharmacokinetic properties of MA90-60 and the structure of the MA9-060: ULK3 complex. Supplementary Figure 5. Pharmacokinetic properties of MA90-60 and the structure of the MA9-060: ULK3 complex. A. Enzymatic inhibition (IC_{50} nM) of BRD4, JAK2, ULK1, ULK2, ULK3 by SG3014 and MA9060. Of note, ULK4 kinase activity assay cannot be calculated, as ULK4 is a pseudokinase [105]. JQ1 is used here as reference for BRD4 inhibitory activity. CQ is used here as a reference control for autophagy inhibition. NA= "no activity". **B.** Microsome stability assay for SG3014 and MA9060, expressed as % of compound remaining after 1 hour incubation with liver microsomes. **C**. X-ray resolved crystal structure of MA9060 (stick model) bound to ULK3 rendered in Pymol (Schrodinger Inc.) at a 2.8 Å resolution. **D**. Table summarizing hULK3-MA9060 crystallographic data collection and refinement statistics. **E.** MA9060 is a dual inhibitor, able to decrease both ULK3 and MYC protein levels in MYC-dependent MM1.S MM cells. JQ1 is here included as the control for BRD4/MYC regulation (**F.**) **G.-H.** MTS assay showed inhibition profile of MA9060 treatment on human mesenchymal stem cells (hMSCs) at 24 (**G**) and 48 **(H**) hours.

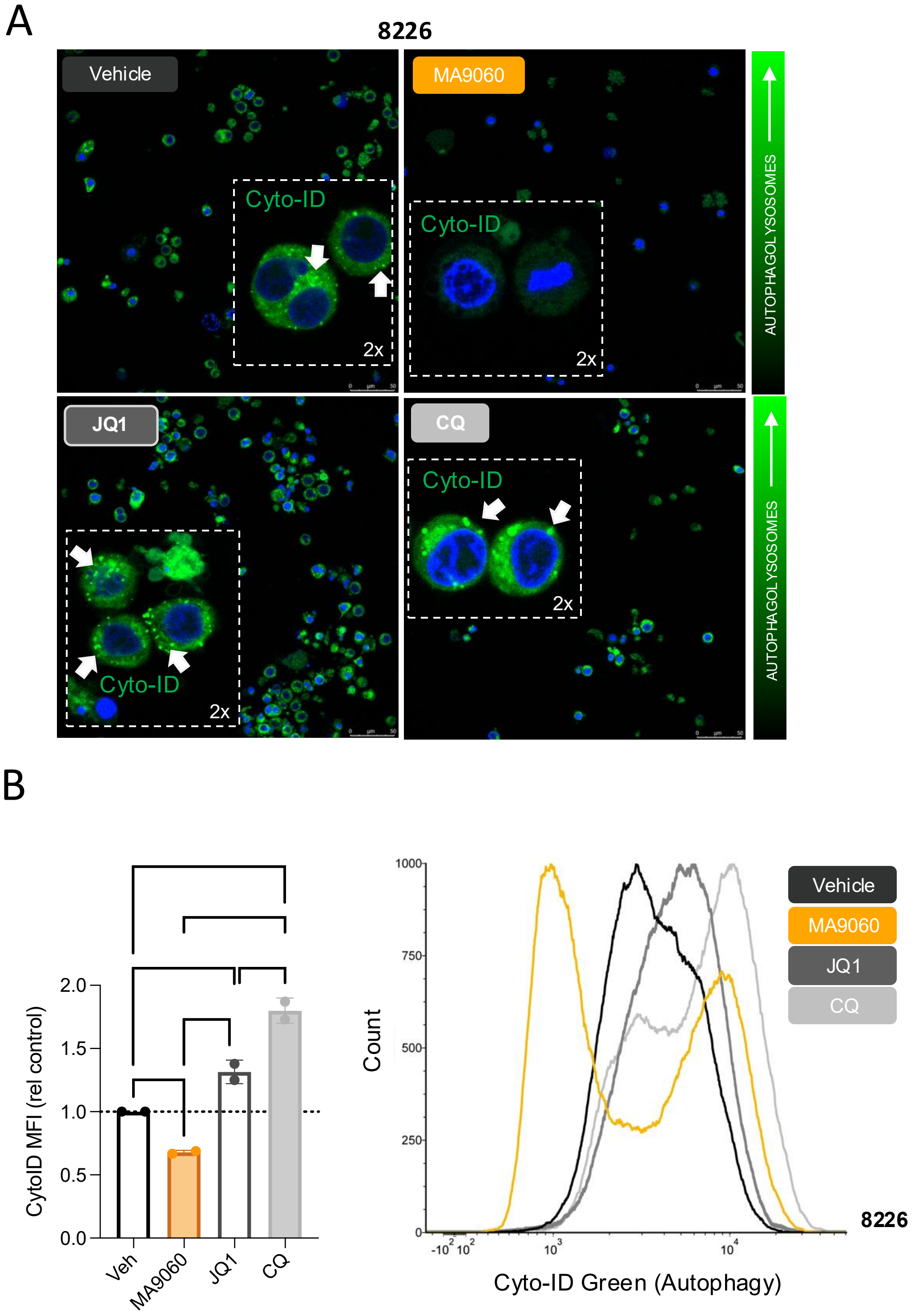

Supplementary Figures - Figure 5

Supplementary Figure 5. **Anti-MM activity of the MA9-060 autophagy inhibitor. A**. Confocal representative images (scale 1-50 µm) and flow cytometry quantification (**B**) of autophagy in 8226 cells, treated with MA9060, JQ1 and CQ measured by a cationic amphiphilic tracer (CAT) autophagic vacuoles dye (CYTO-ID® Autophagy detection kit). Hoechst 33342 dye appears here as the nuclear blue staining. Data is expressed as Cyto ID MFI relative to control (multiple comparisons ANOVA, p <0.0001). Of note, since ULK3 is an upstream initiator of the autophagy pathway, vesicles do not form when an ULK3 inhibitor (MA9-060) is applied. The opposite is true with autophagy flux inhibitor CQ, which allows for the vesicle formation and accumulation yet inhibits the last steps of the pathway.

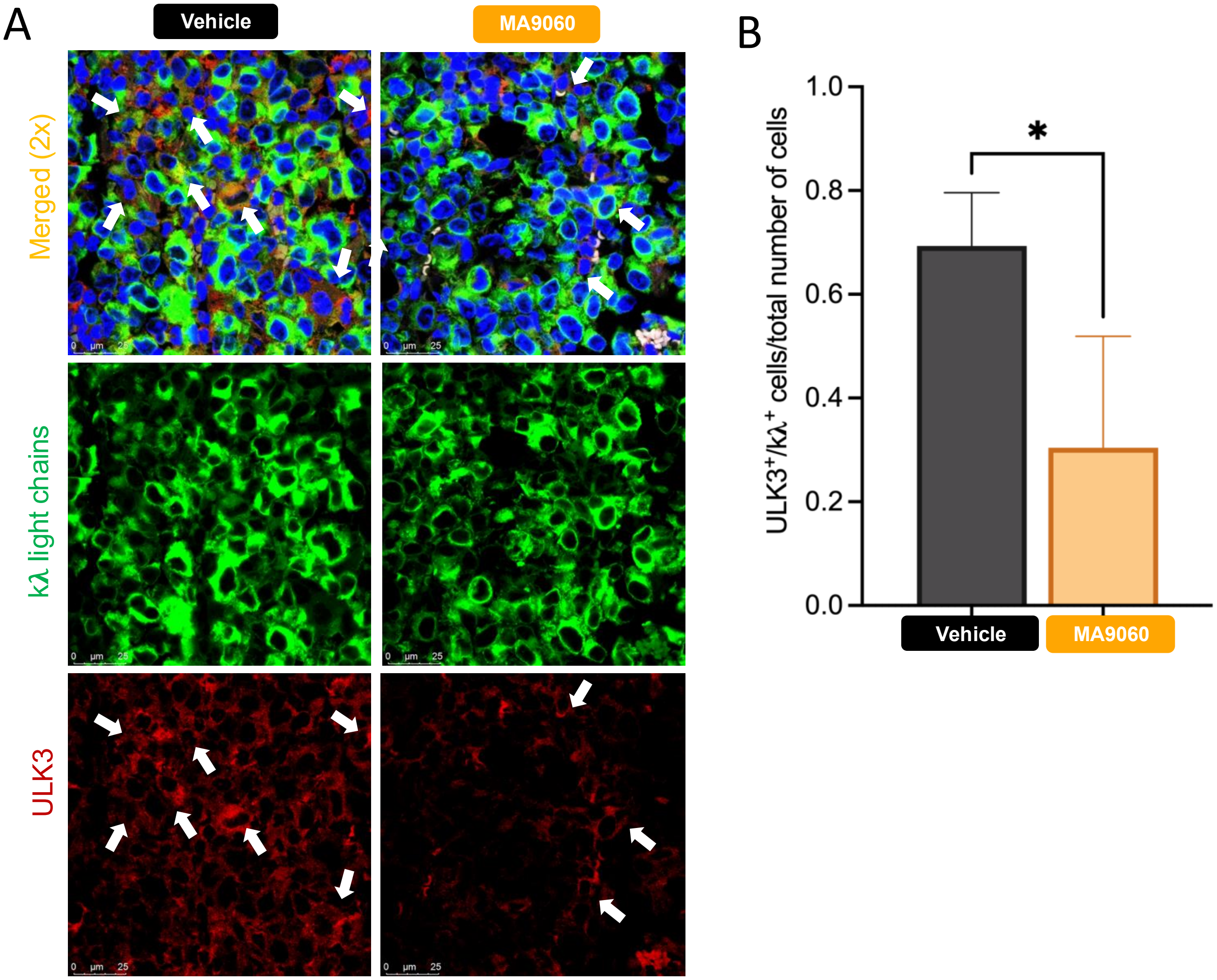

Supplementary Figures - Figure 6

Supplementary Figure 6. MA9060 single agent is effective in reducing ULK3 levels in bone deriving from the U266Luc *in vivo* model. A-B. Representative confocal images (A) and quantification (B) of ULK3+/ kλ light chain+ overlap area, normalized to the total area analyzed (%) of confocal microscopy images (n=5/bone) of formalin fixed, paraffin embedded high limbs, *ex vivo* isolated from U266Luc model, 5 microns sections, blocked in PBS with 10% goat serum and stained for ULK3 (Alexa Fluor® 647 Anti-ULK3 antibody [EPR4888] (CAS# ab310161)) and MM cells (anti-human kλ light chain Dako F0198, F0199 in green) colocalization. Confocal immunofluorescent images were acquired with Leica TCS SP8 system with 40x objective (Leica) and quantified as number of tumor cells positive for ULK3/ total number of cells (Unpaired t test, p=0.0283). IgG and mean of each antibody individually staining were used to determine threshold of positivity.

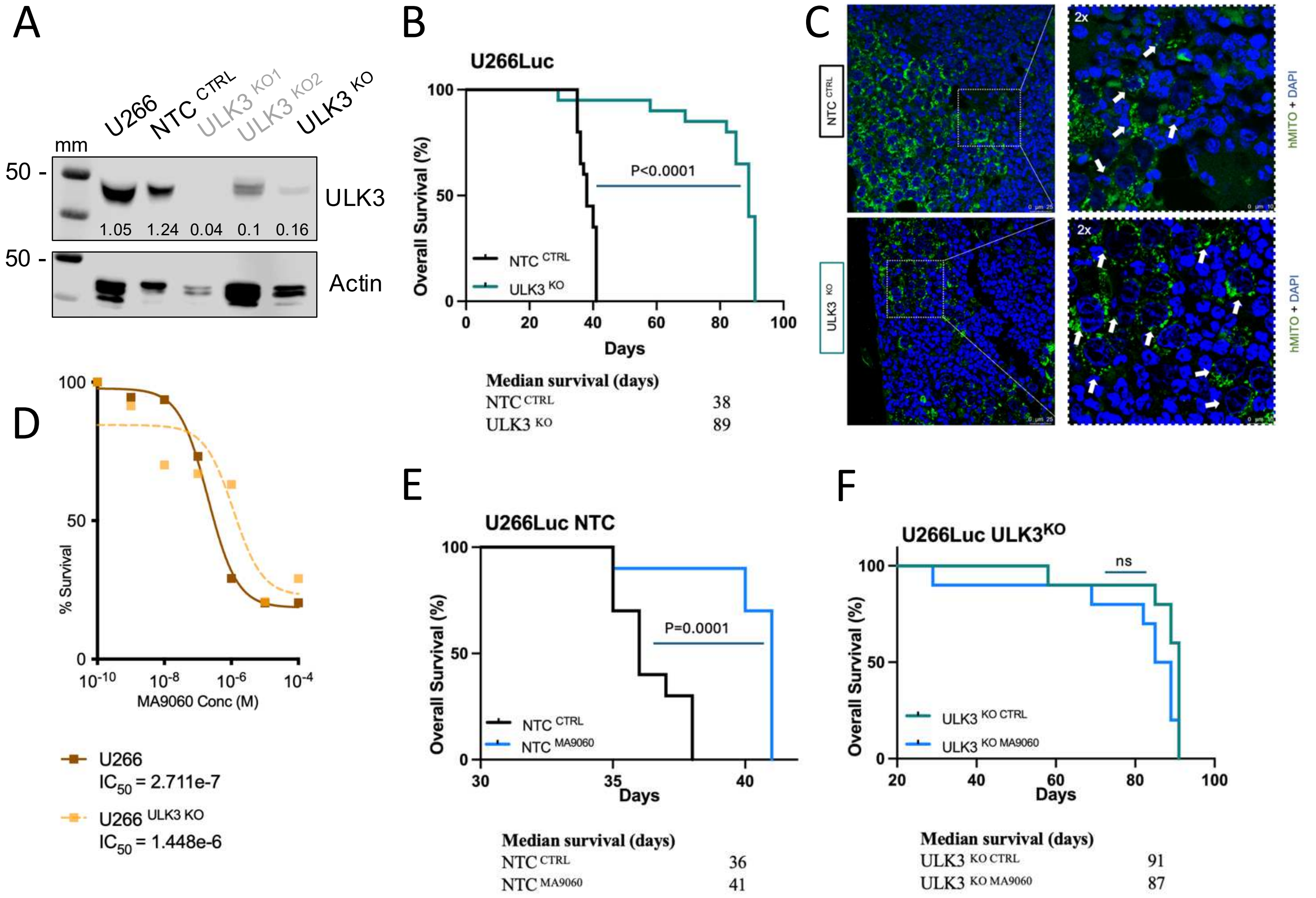

Supplementary Figures - Figure 7

Supplementary Figure 7. ULK3 ablation suppresses MM cell growth. A. Immunoblotting for ULK3 expression in parental, NTC ^{CTRL} and ULK3 ^{KO} U266 cells. Of note, NTC ^{CTRL} and ULK3 ^{KO} were used for the *in vivo ULK3 KO model*. Densitometric quantification values are annotated on the blot. **B.** Mice (*n* = 20/group) were tail vein injected with 1 x10^6 NTC ^{CTRL} and ULK3 ^{KO} U266 MM cells. Mice injected with ULK3 ^{KO} U266 MM cells had a delayed onset of the disease compared to NTC ^{CTRL} . (C) Representative confocal microscopy images of formalin fixed, paraffin embedded tumors (5-micron sections), stained for human mitochondria (hMITO) as a readout of MM cells colocalization. Alexa Fluor Plus 488 goat anti-mouse were used as secondary antibody. (**D**) MTS showing the inhibitory profile of MA9060 on NTC ^{CTRL} and ULK3 ^{KO} for U266 cell line. Treatment with MA9060 (0-100 uM) for 48h show reduced activity of MA9060 in ULK3 KO cell lines. **E.** Mice were divided respectively in two groups, vehicle control (*n = 10*, **β**-HCD Captisol) or MA9-060 (*n = 10)*. Treatment started at day 20 (M-F, 10 mg/kg/day subcutaneously). MA9-060 treatment improves overall survival in the NTC ^{CTRL} arm (median 41 days post-tumor inoculation) compared to vehicle control (CTRL, median 36 days) (**F)** MA9-060 did not affect the overall survival of the group of mice injected with ULK3 ^{KO} U266 MM cells (median 91 days for ULK3 ^{KO} CTRL vs 87 days for MA9-060 treatment). MA9-060 treatment did not show any signs of toxicity (weight loss <20%, ruffled hair) at the chosen dose.

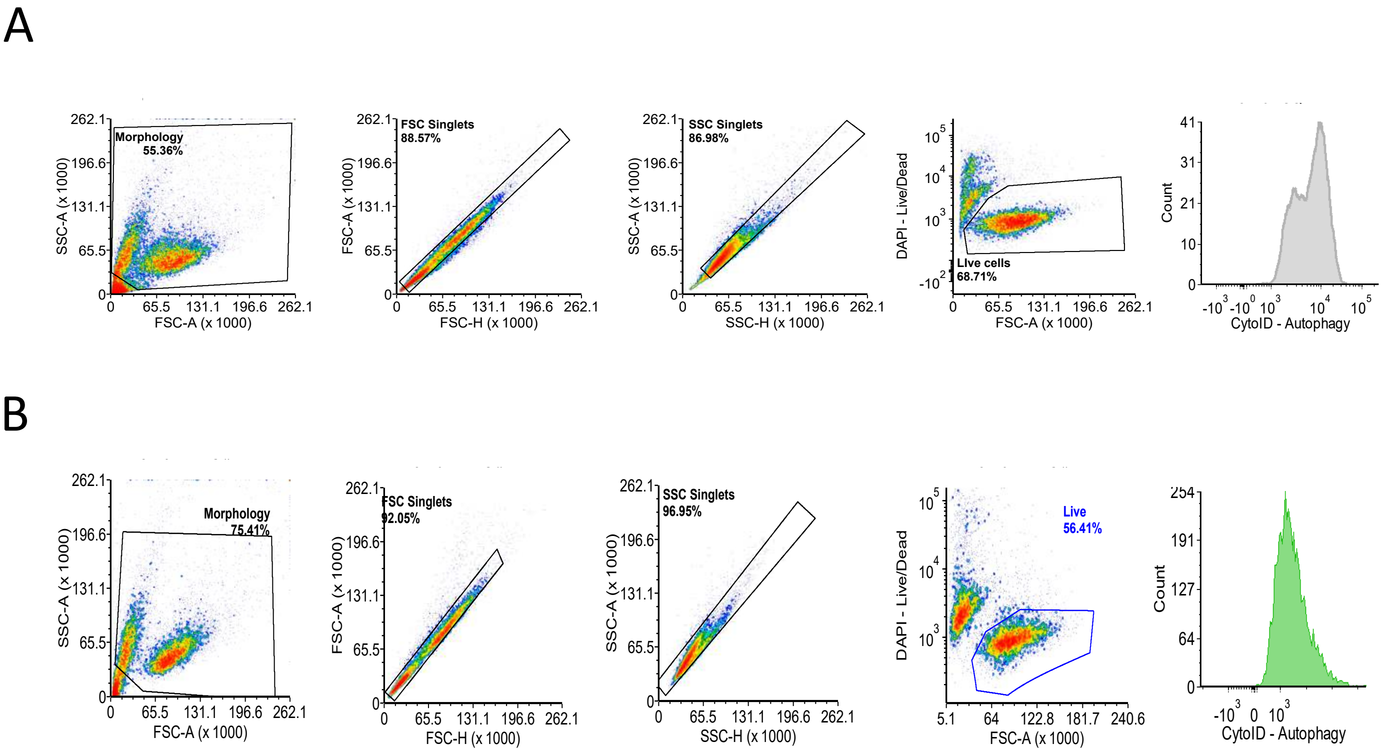

Supplementary Figures - Figure 8

Supplementary Figure 8. Gating strategies used for flow cytometry analysis. A. FSC vs SSC dot plots of MM cells labelled with CYTO-ID marker and DAPI described in Figure 3D and Supplementary Figure 6B. **B.** FSC vs SSC dot plots of MM cells labelled with CYTO-ID marker and DAPI described in Figure 3B.

Means and Medians for Survival Time								
	Mean 95% Confidence Interval				Median 95% Confidence Interval			
group	Estimate	Std. Error	Lower Bound	Upper Bound	Estimate	Std. Error	Lower Bound	Upper Bound
**Vehicle**	60.500	6.702	47.364	73.636	65.000	8.444	48.450	81.550
**MA9060**	108.182	2.844	102.608	113.756	110.000	2.363	105.368	114.632
**JQ1**	92.200	3.617	85.110	99.290	88.000	4.216	79.736	96.264
**CQ**	88.000	4.039	80.084	95.916	86.000	8.521	69.300	102.700
**JQ1+CQ**	98.600	4.375	90.026	107.174	94.000	2.324	89.445	98.555
**Overall**	92.362	2.723	87.025	97.698	94.000	2.542	89.017	98.983

Pairwise Comparisons											
		**Vehicle**		**MA9060**		**JQ1**		**CQ**		**JQ1+CQ**	
		**Chi-Square**	**Sig.**	**Chi-Square**	**Sig.**	**Chi-Square**	**Sig.**	**Chi-Square**	**Sig.**	**Chi-Square**	**Sig.**
**Log Rank (Mantel-Cox)**	**Vehicle**			18.753	1.49E-5	15.617	7.75E-5	12.47	.0004	15.617	7.75E-5
	**MA9060**	18.753	1.49E-5			8.216	.004	12.035	.001	1.980	.159
	**JQ1**	15.617	7.75E-5	8.22	.004			.502	.479	1.554	.213
	**CQ**	12.470	.0004	12.035	.001	.502	.479			2.316	.128
	**JQ1+CQ**	15.617	7.75E-5	1.98	.159	1.554	.213	2.316	.128		

Overall Comparisons			
	**Chi-Square**	**df**	**Sig.**
**Log Rank (Mantel-Cox)**	56.696	4	1.43E-11
**Breslow (Generalized Wilcoxon)**	55.174	4	2.99E-11
**Tarone- Ware**	56.129	4	1.88E-11

Test of equality of survival distributions for the different levels of group.

Supplementary Figures - Figure 9

Supplementary Figure 9. U266 *in vivo* model: statistical analysis. Statistical analysis tables of Means and Medians for survival time, Pairwise comparisons among groups (Chi square) and significance, and overall comparison.

Main Figure 1E	B Cells	U266	8226
ULK1	0.005	0.04	0.01
ULK3	0.007	0.1	0.1

Main Figure 2A-E	U266 WT	U266 KD ULK3	MM1.S WT	MM1.S KD ULK3	WM1366 WT	WM1366 KD ULK3
ULK3	4.38	0.85	3.39	1.68	0.56	0.13
LC3I	0.5	0.64	0.41	0.93	0.76	1.79
LC3II	0.07	0.09	0.04	0.16	0.13	0.29

Main Figure 3B	CTRL	MA9060	JQ1	CQ
ULK3	15.17	3.33	16.81	11.1
ULK1	7.21	2.44	6.20	0.78
LC3I	3.01	1.78	3.36	1.69
LC3II	0.34	0.10	0.40	0.14

Main Figure 6D	U266	U266-PSR	8226	8226-B25
ULK3	0.19	0.84	0.26	0.76

Main Figure 7H	8226	U266	Pt971	Pt1050 CTRL	Pt1050 MA9 2h	Pt1050 MA9 6h
ULK3	0.09	0.08	0.01	0.03	0.03	0.03
ULK1	0.07	0.12	0.35	0.28	0.67	0.35

Supplementary Figures - Figure 10

Supplementary Figure 10. Quantification of immunoblots included in Main Figures. Indicated values represent the raw data of the signal quantification normalized to actin.

Suppl. Figure 4C	SG3014 0	SG3014 0.03	SG3014 0.1	SG3014 0.3	SG3014 1	SG3014 3
ULK1	0.02	0.01	0.03	0.11	0.12	0.1
ULK3	0	0.05	0.07	0.27	0.47	0.44

Suppl. Figure 4D	JQ1 0	JQ1 0.03	JQ1 0.1	JQ1 0.3	JQ1 1	JQ1 3
ULK1	0.11	0.08	0.14	0.1	0.23	0.11
ULK3	0.07	0.15	0.22	0.2	0.15	0.1

Suppl. Figure 4E	CTRL	SG3014	JQ1	CQ	TEMS
ULK3	12.27	4.56	10.32	2.09	11.13
ULK1	0.0007	0.000	0.0004	0	0.0007
LC3I	0.23	0.3	0.15	0.22	0.14
LC3II	0.03	0.08	0.03	0.15	0.02

Suppl. Figure 5E	MA9060 0	MA9060 0.03	MA9060 0.1	MA9060 0.3	MA9060 1	MA9060 3
ULK1	7.96	7.45	7.71	7.40	7.56	7.70
ULK3	9.02	9.71	8.48	5.77	5.93	3.51
MYC	50	49.08	27.24	13.25	10.8	4.17

Suppl. Figure 5F	JQ1 0	JQ1 0.03	JQ1 0.1	JQ1 0.3	JQ1 1	JQ1 3
ULK1	0.02	0.02	0.02	0.03	0.01	0.01
ULK3	0.07	0.09	0.06	0.06	0.04	0.05
MYC	0.34	0.35	0.25	0.14	0.05	0.04

Supplementary Figure 11. Quantification of immunoblots included in Supplementary Figures. Indicated values represent the raw data of the signal quantification normalized to actin/vinculin.

Category	Number (percent)
**Age**, year, median (range)	64 (33-88)
**Gender**	
Male	453 (56%)
Female	360 (44%)
**Race**	
White	699 (86%)
Black	82 (10%)
Other race & Unknown	32 (4%)
**Ethnicity**	
Non-Spanish	746 (92%)
Latino	64 (8%)
Unknown	3 (0%)
**Disease State**	
MGUS	64 (8%)
SMOL	57 (7%)
NDMM	207 (25%)
ERMM	303 (37%)
LRMM	182 (22%)
**Mayo Risk 2018 (SMM only)**	
Low	15 (26%)
Intermediate	23 (40%)
High	18 (32%)
Unknown	1 (2%)
**ISS (NDMM, ERMM, LRMM - at diagnosis)**	
I	269 (39%)
II	166 (24%)
III	118 (17%)
NA	139 (20%)
**R-ISS (NDMM, ERMM, LRMM - at diagnosis)**	
I	115 (17%)
II	315 (46%)
III	63 (9%)
NA	199 (29%)
**FISH/Cytogenetics**	
Amp/Gain(1q21)	295 (36%)
Del(1p)	30 (4%)
Del(13q)	316 (39%)
Del(17p)	115 (14%)
t(4;14)	65 (8%)
t(11;14)	147 (18%)
t(14;16)	23 (3%)
FISH not available	51 (6%)
**Risk assessment**	
Standard Risk	395 (52%)
Intermediate Risk*	185 (24%)
High Risk**	182 (24%)
Double hit***	111 (15%)
Triple hit****	10 (1%)

Supplementary Table 1

Supplementary Table 1. Patient's demographics of multiple myeloma study population. Patient's demographics characteristics of multiple myeloma patients included in this study. Age, gender, race, ethnicity, disease state, Mayo Risk 2028, ISS, R-ISS and FISH/Cytogenetics classification, as well as risk assessment, are here indicated as percent. *Intermediate risk defined as exhibiting Amp/Gain(1q21) and NOT high-risk features. **High risk defined as exhibiting Del(17p) and/or t(4;14), & t(14;16). ***High risk samples presenting two high risk features and/or Amp/Gain(1q21). ***High risk samples presenting three high risk features and/or Amp/Gain(1q21).

Patient_ID	Disease Status	BTZ	CFZ	MA9060	JQ1+CQ	CFZ+ MA9060	CFZ+ JQ1+CQ	Additive CFZ+MA9060	Additive CFZ+JQ1+CQ
Pt693	SMOL	87.905	97.333	70.973		70.133		69.102	
Pt718	SMOL	53.735	58.310	76.172		63.024		49.384	
Pt740	SMOL	32.547	45.352	62.223		27.004		34.833	
Pt899	SMOL	59.818	53.140	28.290	85.045	23.245	36.555	22.990	48.435
Pt737	MGUS	35.864	45.483	42.854		21.791		28.571	
Pt912	PCL	26.800	23.632	41.302	43.457	15.019	19.382	15.473	18.153
Pt672	NDMM	45.751	56.496	49.156		43.682		33.055	
Pt677	NDMM	30.916	38.487	43.301		25.754		28.018	
Pt677	NDMM	33.959	37.936	45.593		25.523		27.757	
Pt689	NDMM	40.257	43.578	97.182		43.271		42.688	
Pt694	NDMM	31.313	49.029	47.506		29.294		30.986	
Pt697	NDMM	44.112	45.229	65.916		30.023		39.143	
Pt700	NDMM	42.763	48.455	71.574		43.744		40.255	
Pt700	NDMM	44.093	54.337	71.393		47.268		43.857	
Pt738	NDMM	46.001	52.453	65.975		44.896		40.847	
Pt745	NDMM	47.650	68.911	55.970		40.861		45.268	
Pt852	NDMM	23.262	28.629	41.956	81.865	31.119	29.032	21.058	26.819
Pt856	NDMM	36.749	48.272	72.334	92.310	53.682	46.284	39.555	46.054
Pt866	NDMM	36.635	75.161	37.556	82.294	31.831	41.042	32.527	64.893
Pt875	NDMM	45.303	41.904	21.103	62.655	14.892	32.693	13.838	30.287
Pt885	NDMM	54.295	51.382	27.776	75.600	20.755	33.098	20.892	42.345
Pt885	NDMM	53.664	45.860	28.777	78.798	21.725	33.656	20.659	39.600
Pt906	NDMM	59.784	58.079	43.689	96.251	28.931	43.015	37.465	57.580
Pt935	NDMM	31.629	46.326	25.467	74.184	18.614	34.837	18.677	39.942
Pt937	NDMM	46.111	51.703	48.139	79.570	49.940	46.917	31.743	44.734
Pt937	NDMM	46.768	49.814	40.693	77.446	38.279	36.852	29.270	43.313
Pt956	NDMM	64.107	68.610	67.208	73.282	50.188	51.397	48.270	52.141
Pt956	NDMM	61.177	64.376	74.021	76.778	61.064	54.985	51.288	52.447
Pt961	NDMM	70.805	64.332	66.118	85.750	74.044	61.319	43.635	56.851
Pt961	NDMM	67.849	67.807	49.072	75.939	58.337	47.773	36.288	53.866
Pt669	ERMM	37.041	48.580	29.511		30.559		19.608	
Pt680	ERMM	39.728	43.762	35.236		23.678		27.061	
Pt680	ERMM	39.652	43.248	42.605		28.890		29.324	
Pt685	ERMM	34.249	44.316	100.000		45.439		44.316	
Pt853	ERMM	63.604	62.247	49.879	82.124	33.421	46.099	40.048	54.109
Pt853	ERMM	64.834	58.956	54.514	81.999	40.629	41.338	41.738	48.575
Pt951	ERMM	57.542	62.878	62.382	86.882	59.621	53.765	43.822	57.018
Pt648	LRMM	48.724	48.435	42.835		31.387		31.841	
Pt652	LRMM	65.980	67.924	100.000		93.777		67.924	
Pt653	LRMM	54.219	60.160	41.560		34.309		32.922	
Pt655	LRMM	29.968	37.063	26.032		22.090		20.480	
Pt663	LRMM	45.417	48.477	47.016		33.218		35.235	
Pt665	LRMM	47.552	52.826	70.141		66.744		38.507	
Pt666	LRMM	37.470	40.984	49.231		30.496		29.108	
Pt691	LRMM	38.965	40.781	68.663		36.593		33.758	
Pt695	LRMM	46.540	54.535	70.638		47.622		43.886	
Pt707	LRMM	60.814	60.754	73.564		36.909		48.846	
Pt865	LRMM	54.103	60.759	59.277	71.908	52.587	41.351	41.244	48.423
Pt888	LRMM	100.000	51.003	59.977	96.068	35.102	40.500	37.246	50.231
Pt893	LRMM	100.000	92.226	96.916	88.437	93.200	96.174	89.389	82.285
Pt909	LRMM	29.196	32.841	46.043	66.249	11.666	25.239	25.931	30.021
Pt917	LRMM	78.984	75.009	73.927	94.334	75.941	68.040	55.786	70.815
Pt920	LRMM	42.040	39.820	44.399	76.070	42.616	31.795	25.232	35.667
Pt923	LRMM	45.019	54.728	28.337	73.221	28.817	37.417	23.882	45.744
Pt932	LRMM	28.145	43.433	45.003	80.718	28.113	38.613	28.096	39.511
Pt933	LRMM	39.573	54.495	57.893	61.428	42.481	33.236	38.564	39.276
Pt936	LRMM	45.292	50.956	83.700	69.654	80.066	41.137	43.539	40.161
Pt940	LRMM	43.043	63.001	72.923	55.127	57.193	39.470	46.257	38.736
Pt945	LRMM	55.241	53.027	39.091	71.366	32.799	40.008	26.224	41.333
Pt948	LRMM	58.089	57.940	24.690	82.584	25.315	40.537	20.703	50.632
Pt957	LRMM	82.570	82.913	80.043	100.000	58.561	92.998	66.569	82.913

AUC

Supplementary Table 2

Supplementary Table 2. AUC as a predictor of ex-vivo drug efficacy for MM patients at different stages of the disease. *Ex vivo* EMMA platform assays were used to quantify the chemosensitivity of primary MM cells (CD138 selection) plated in a collagen matrix with bone marrow stroma and patient plasma. Tumor cells were treated with Carfilzomib (CFZ), MA9-060, JQ1, CQ, and combination of them for 96 hours (EVOS FL Auto, Thermo Fisher Scientific, Waltham, MA). Synergy was determined by using the method described by Sudalagunta et al [99]. Area under the curve (AUC) is shown as a metric of drug sensitivity (< AUC = > EFFICACY).

Patient_ID	Disease Status	BTZ	CFZ	MA9060	JQ1+CQ	CFZ+ MA9060	CFZ+ JQ1+CQ	Additive CFZ+MA9060	Additive CFZ+JQ1+CQ
Pt693	SMOL			1.55E-05		3.09E-08		2.38E-08	
Pt718	SMOL	1.82E-09	1.32E-09	4.43E-06		4.67E-10		1.11E-09	
Pt740	SMOL	3.42E-10	4.17E-09	3.17E-06		1.46E-09		2.43E-09	
Pt899	SMOL	6.16E-09	5.80E-09	3.65E-07		3.24E-10	1.55E-09	9.12E-10	3.21E-09
Pt912	PCL	1.78E-09	1.00E-09	1.36E-07	2.34E-07	1.30E-09	4.72E-10	3.19E-10	4.29E-10
Pt737	MGUS	2.04E-09	3.02E-09	2.20E-06		1.27E-09		2.25E-09	
Pt672	NDMM	1.96E-09	4.32E-09	8.92E-07		1.59E-09		9.54E-10	
Pt677	NDMM	1.44E-09	2.09E-09	4.64E-07		7.08E-10		9.15E-10	
Pt677	NDMM	1.99E-09	1.76E-09	5.92E-07		9.38E-10		9.12E-10	
Pt689	NDMM	2.23E-09	2.74E-09			2.59E-09		2.63E-09	
Pt694	NDMM	1.56E-09	4.83E-09	2.22E-06		1.45E-09		2.69E-09	
Pt697	NDMM	3.15E-09	3.46E-09	3.01E-06		1.98E-09		2.71E-09	
Pt700	NDMM	2.21E-09	3.16E-09	6.19E-06		1.48E-09		1.90E-09	
Pt700	NDMM	1.90E-09	3.03E-09	5.66E-06		1.52E-09		1.90E-09	
Pt738	NDMM	3.39E-09	3.77E-09	2.85E-06		1.16E-09		1.71E-09	
Pt745	NDMM	6.64E-09	1.85E-08	3.92E-06		4.27E-09		8.40E-09	
Pt852	NDMM	7.66E-10	1.01E-09	2.13E-07	1.13E-05	3.13E-10	6.54E-10	4.34E-10	8.13E-10
Pt856	NDMM	3.33E-09	3.28E-09	7.45E-06		1.93E-09	1.62E-09	2.14E-09	3.04E-09
Pt866	NDMM	1.19E-09	2.41E-08	7.07E-07	9.59E-06	1.93E-09	3.48E-09	1.76E-09	9.15E-09
Pt875	NDMM	2.17E-09	6.06E-10	3.37E-07	1.04E-06	9.96E-10	3.15E-10	3.67E-10	3.73E-10
Pt885	NDMM	7.68E-09	4.45E-09	3.29E-07	9.96E-06	6.75E-10	1.26E-09	5.10E-10	1.45E-09
Pt885	NDMM	7.21E-09	2.36E-09	2.76E-07		8.45E-10	1.46E-09	6.24E-10	1.43E-09
Pt906	NDMM	3.42E-09	3.71E-09	1.14E-06		1.64E-09	1.53E-09	1.97E-09	3.26E-09
Pt935	NDMM	1.05E-09	3.38E-09	1.40E-07	4.90E-06	8.07E-10	1.89E-09	3.38E-10	1.55E-09
Pt937	NDMM	2.67E-09	3.77E-09	5.55E-07	1.85E-05	1.02E-09	1.20E-09	6.60E-10	1.21E-09
Pt937	NDMM	2.94E-09	1.83E-09	5.21E-07	4.51E-06	6.22E-10	5.99E-10	6.80E-10	6.28E-10
Pt956	NDMM	6.68E-09	1.88E-08	6.97E-06		2.05E-09	4.94E-10	3.90E-09	1.14E-09
Pt956	NDMM	6.05E-09	7.38E-09	8.28E-06	1.87E-05	1.03E-08	2.44E-09	5.82E-09	1.80E-09
Pt961	NDMM	3.96E-08	2.30E-09			4.52E-08	2.59E-09	4.17E-10	4.79E-10
Pt961	NDMM	1.16E-08	1.49E-08	1.95E-07	7.88E-06	6.32E-09	6.71E-10	4.26E-10	2.19E-09
Pt669	ERMM	2.88E-09	3.69E-09	3.66E-07		1.03E-09		5.62E-10	
Pt680	ERMM	2.10E-09	2.14E-09	1.07E-06		5.40E-10		1.11E-09	
Pt680	ERMM	2.44E-09	2.46E-09	9.52E-07		7.33E-10		1.02E-09	
Pt685	ERMM	1.54E-09	2.85E-09			2.57E-09		2.85E-09	
Pt853	ERMM	4.85E-09	6.67E-09	2.74E-06		2.26E-09	3.16E-09	3.33E-09	4.34E-09
Pt853	ERMM	6.71E-09	5.91E-09	2.71E-06		2.26E-09	2.43E-09	3.18E-09	4.30E-09
Pt951	ERMM	7.28E-09	8.37E-09	2.12E-06		3.86E-09	2.92E-09	2.31E-09	3.81E-09
Pt648	LRMM	3.02E-09	2.85E-09	2.51E-06		1.91E-09		2.85E-09	
Pt652	LRMM	1.02E-08	1.40E-08					1.40E-08	
Pt653	LRMM	3.63E-09	7.46E-09	1.15E-06		1.51E-09		2.17E-09	
Pt655	LRMM	1.97E-09	2.52E-09	7.63E-07		6.55E-10		1.62E-09	
Pt663	LRMM	3.84E-09	4.19E-09	2.05E-06		2.02E-09		2.66E-09	
Pt665	LRMM	2.13E-09	7.29E-09			4.13E-08		1.08E-09	
Pt666	LRMM	2.13E-09	2.88E-09	1.64E-06		9.98E-10		1.46E-09	
Pt691	LRMM	3.71E-09	2.72E-09	4.72E-06		1.27E-09		2.01E-09	
Pt695	LRMM	2.17E-09	4.78E-09	7.18E-06		1.91E-09		3.11E-09	
Pt707	LRMM	5.96E-09	4.20E-09	6.25E-06		2.45E-09		1.81E-09	
Pt865	LRMM	4.76E-09	5.81E-09	1.60E-06	7.19E-06	2.57E-09	1.58E-09	2.00E-09	3.45E-09
Pt888	LRMM		3.99E-09	4.05E-06		1.38E-09	1.06E-09	3.04E-09	3.95E-09
Pt893	LRMM								3.85E-08
Pt909	LRMM	1.93E-09	8.21E-10	6.77E-07	1.56E-06	2.68E-10	4.95E-10	7.24E-10	7.08E-10
Pt917	LRMM						2.46E-08	2.17E-08	
Pt920	LRMM	2.82E-09	1.67E-09	5.49E-07	3.29E-06	8.67E-09	5.09E-10	7.76E-10	1.04E-09
Pt923	LRMM	3.67E-09	5.97E-09	3.39E-07	3.23E-06	9.63E-10	1.39E-09	8.42E-10	2.71E-09
Pt932	LRMM	1.44E-09	2.53E-09	3.76E-07	1.92E-06	9.23E-10	9.42E-10	6.62E-10	9.75E-10
Pt933	LRMM	4.38E-09	5.69E-09	3.09E-06	4.76E-07	4.75E-10	4.47E-10	3.10E-09	8.64E-10
Pt936	LRMM	3.76E-09	7.00E-09		3.51E-06		8.11E-10	4.32E-09	1.71E-09
Pt940	LRMM	1.93E-09	6.61E-09		2.39E-07		4.76E-10	3.56E-09	5.97E-10
Pt945	LRMM	7.73E-09	5.88E-09	3.68E-07	8.01E-06	5.86E-10	1.90E-09	8.10E-10	2.07E-09
Pt948	LRMM	2.98E-09	2.41E-09	4.08E-07		3.44E-10	1.85E-09	7.41E-10	1.41E-09
Pt957	LRMM					1.41E-08			

LD_{50}

Supplementary Table 3

Supplementary Table 3. Lethal Dose 50 (LD_{50}) as a predictor of ex-vivo drug efficacy for MM patients at different stages of the disease. *Ex vivo* EMMA platform assays were used to quantify the chemosensitivity of primary MM cells (CD138 selection) plated in a collagen matrix with bone marrow stroma and patient plasma. Tumor cells were treated with Carfilzomib (CFZ), MA9-060, JQ1, CQ, and combination of them for 96 hours (EVOS FL Auto, Thermo Fisher Scientific, Waltham, MA). Synergy was determined by using the method described by Sudalagunta et al [99]. Lethal Dose 50 (LD_{50}) is shown as a metric of drug sensitivity (< LD_{50} = > EFFICACY).

Patient_ID	Disease Status	MA9_AUC	CFZ_AUC	*ULK3*_Exp	*ULK1*_Exp	*BRD4*_Exp
Pt718	SMOL	76.1715915	58.3103315	1.1111	1.6188	1.4275
Pt875	NDMM	21.1030514	41.9036325	0.11632	1.4532	2.3898
Pt694	NDMM	47.505789	49.0287275	0.14407	1.4709	0.56377
Pt738	NDMM	65.9745538	52.4528299	0.40985	1.7344	1.629
Pt951	NDMM	62.381599	62.8781243	0.66191	1.8228	0.94922
Pt700	NDMM	71.3930512	54.3373609	0.97187	1.7589	1.2525
Pt856	NDMM	72.3344888	48.2721978	1.1273	1.3721	1.6467
Pt825	NDMM	100	59.8643296	1.2129	1.7609	1.263
Pt639	NDMM	59.5580305	52.9675661	1.2523	0.46477	-2.1074
Pt625	NDMM	26.0809396	36.2526823	1.2792	0.29877	-1.4403
Pt885	NDMM	27.7764237	51.3821507	1.3137	1.1765	1.8455
Pt693	NDMM	70.9729106	97.3329689	1.4582	1.5322	1.2148
Pt906	NDMM	43.6886874	58.0793486	1.7538	1.4031	1.4366
Pt866	NDMM	37.5564632	75.1609617	1.8198	1.1952	1.021
Pt821	NDMM	58.3986518	54.7881937	1.8677	1.6205	1.7501
Pt697	NDMM	65.9158977	45.2289007	2.0677	1.2003	1.0179
Pt638	NDMM	48.2296733	44.5200218	2.1092	1.1081	-1.7116
Pt672	NDMM	49.1561364	56.4961152	2.718	2.0249	1.5308
Pt620	NDMM	46.8353149	36.7755583	3.2278	3.7769	3.1719
Pt935	NDMM	25.467269	46.3261432	1.4949	1.3284	1.0877
Pt830	ERRMM	58.8826954	61.4087523	0.20403	1.2551	0.66307
Pt669	ERRMM	29.5112298	48.5798798	1.453	2.2697	1.5344
Pt822	ERRMM	58.9631405	62.961258	1.4637	1.8106	1.4216
Pt680	ERRMM	35.2362103	43.7622193	1.9707	1.4896	-1.4224
Pt636	ERRMM	50.2967868	53.5205305	2.1911	-0.10263	-1.895
Pt695	LRRMM	70.6384233	54.5349512	0.70609	1.8856	1.5212
Pt948	LRRMM	24.6902914	57.9397996	0.82174	1.8068	1.5887
Pt765	LRRMM	69.3138334	59.7692816	0.96297	0.86345	1.241
Pt685	LRRMM	100	44.3161737	1.2444	1.3898	1.497
Pt849	LRRMM	66.578531	99.0559494	1.3497	1.394	1.0777
Pt933	LRRMM	57.8933071	54.4949444	1.3996	2.0027	1.5861
Pt707	LRRMM	73.5642851	60.7540003	1.428	1.7474	2.1464
Pt808	LRRMM	79.8561192	47.1145526	1.4516	1.6356	1.0673
Pt773	LRRMM	47.3947367	32.9906615	1.4931	0.55064	1.146
Pt691	LRRMM	68.6628027	40.7805587	1.5561	1.2366	1.9537
Pt655	LRRMM	26.032317	37.0632006	1.5958	1.1512	0.66219
Pt888	LRRMM	59.9769283	51.0033148	1.5995	1.5438	1.2612
Pt665	LRRMM	70.141223	52.8264049	1.6209	1.9261	-0.68891
Pt923	LRRMM	28.3372434	54.7279644	1.6777	2.0719	1.5697
Pt622	LRRMM	21.989338	32.8938952	1.9223	0.3823	-1.6486
Pt865	LRRMM	59.2772579	60.7589688	1.9448	2.1786	2.4273
Pt814	LRRMM	68.9071944	62.2341467	1.9471	1.8957	1.6785
Pt663	LRRMM	47.0156972	48.4766194	2.2256	-1.1208	-2.5812
Pt666	LRRMM	49.2313403	40.9838082	2.5508	1.9232	2.4299

Supplementary Table 4

Supplementary Table 4. RNA-sequencing analyses levels of *BRD4*, *ULK1* and *ULK3* expression in MM patients at different stages of the disease. RNA-sequencing of fresh bone marrow (BM) aspirate cells enriched for CD138 (Moffitt cohort & MCC17814) was performed and RNA expression values are here reported as normalized Z-score values of single genes *ULK1*, *ULK3*, *BRD4*.

Protein/Name	Manufacturer	Catalogue Number	Dilution	Application
ULK3	Abcam	124947	1:1000	WB/IF
ULK3	MyBioSource	MBS9200567	1:500	IF
P-ATG13 (S355)	Cell Signaling	26839S	1:1000	WB
ATG13 (E1Y9V)	Cell Signaling	13468S	1:1000	WB
Mouse anti-Human ATG13	MyBioSource	MBS8564879	1:1000	WB/IP
FIP200	Cell Signaling	12436S	1:1000	WB
ULK1	Proteintech	20986-1-AP	1:1000	WB
LC3I/II	Cell Signaling	12741S	1:1000	WB
Actin	SantaCruz	Sc-47778	1:3000	WB
MYC	Abcam	32072	1:1000	WB
vinculin	Cell Signaling	13901	1:1000	WB
Anti-human Kappa light chain	DAKO	F0198	1:500	IF
Anti-human Lambda light chain	DAKO	F0199	1:500	IF
Anti-human mitochondria	Millipore	MAB1273	1:300	IF
Ki67	Cell Signaling	9129S	1:500	IF
Cleaved caspase 3	Cell Signaling	9664L	1:300	IF
Rabbit - IgG	Invitrogen	32135	1:200	IF
IgG1 Isotype Control	Millipore Sigma	M5284	1:200	IF
Alexa Fluor647 anti-ULK3 [EPR4888]	Abcam	ab310161	1:50	IF
Alexa Fluor Plus 488 goat anti-mouse	Invitrogen	A32723	1:1000	IF

Supplementary Table 5

Supplementary Table 5. Antibodies used in these studies. Details about protein name, manufacturer, catalogue number, dilution used and application of antibodies.

Disease Status	RNA_ID										
MGUS	SL233336	SL233357	SL235855	SL240814	SL240882	SL240916	SL242337	SL253601	SL310722	SL390324	SL404304	
	SL233340	SL235833	SL238872	SL240827	SL240887	SL240925	SL242357	SL258023	SL322886	SL390348	SL404330	
	SL233353	SL235835	SL238880	SL240831	SL240901	SL240933	SL242381	SL264936	SL322901	SL399340	SL405582	
	SL233354	SL235840	SL240802	SL240834	SL240904	SL240936	SL242383	SL264944	SL373319	SL399351	SL405583	
	SL233355	SL235850	SL240804	SL240836	SL240913	SL242322	SL253597	SL264958	SL373322	SL399352		
	SL233356	SL235851	SL240808	SL240879	SL240914	SL242325	SL253599	SL310702	SL375743	SL404277		
SMOL	SL204935	SL219812	SL233359	SL235843	SL240793	SL240849	SL240877	SL240946	SL258046	SL271849	SL391457	SL405607
	SL215230	SL219819	SL233362	SL238876	SL240796	SL240850	SL240897	SL240949	SL264892	SL322900	SL399344	SL405730
	SL215249	SL233334	SL235836	SL238892	SL240806	SL240853	SL240912	SL242321	SL264917	SL340699	SL399347	
	SL217493	SL233339	SL235837	SL240774	SL240809	SL240856	SL240920	SL242370	SL264968	SL375742	SL404305	
	SL219799	SL233351	SL235842	SL240780	SL240820	SL240858	SL240931	SL242372	SL271846	SL375778	SL404325	
NDMM	SL204930	SL215245	SL217471	SL218333	SL219814	SL240842	SL242400	SL271840	SL340738	SL375750	SL391484	
	SL204932	SL215247	SL217472	SL218341	SL219815	SL240852	SL242402	SL271848	SL340740	SL375751	SL399173	
	SL204937	SL215248	SL217473	SL218342	SL219817	SL240860	SL258026	SL271879	SL340765	SL375749	SL399197	
	SL204938	SL215250	SL217474	SL218344	SL219820	SL240864	SL258043	SL271902	SL373323	SL375752	SL399201	
	SL204940	SL215253	SL217478	SL218355	SL233365	SL240871	SL258048	SL271915	SL373325	SL375765	SL399204	
	SL204942	SL215254	SL217483	SL218356	SL235839	SL240898	SL264890	SL271916	SL373333	SL375780	SL399317	
	SL204944	SL215255	SL217484	SL218366	SL235865	SL240900	SL264937	SL271938	SL373343	SL375782	SL399324	
	SL204946	SL215257	SL217490	SL218367	SL238881	SL240902	SL264939	SL278537	SL373349	SL375783	SL399325	
	SL215226	SL215258	SL217491	SL218368	SL238891	SL240923	SL264940	SL310717	SL373352	SL375784	SL399326	
	SL215227	SL215264	SL217492	SL218369	SL240786	SL240928	SL264941	SL310721	SL373353	SL375786	SL399327	
	SL215229	SL215265	SL217495	SL219801	SL240797	SL240938	SL264946	SL310723	SL373354	SL390298	SL404331	
	SL215233	SL215266	SL217497	SL219802	SL240798	SL240940	SL264947	SL316615	SL373358	SL390322	SL405557	
	SL215234	SL215268	SL217503	SL219804	SL240799	SL240947	SL264960	SL322903	SL373368	SL390323	SL405581	
	SL215235	SL215269	SL217506	SL219806	SL240810	SL240948	SL264961	SL322929	SL373370	SL390344	SL405631	
	SL215237	SL215270	SL217509	SL219808	SL240817	SL240960	SL264962	SL340698	SL373372	SL390346	SL405725	
	SL215239	SL215271	SL217510	SL219809	SL240819	SL242310	SL264964	SL340700	SL373374	SL390347	SL405728	
	SL215241	SL217468	SL218329	SL219810	SL240821	SL242333	SL264967	SL340701	SL373376	SL391434	SL405731	
	SL215242	SL217469	SL218330	SL219811	SL240833	SL242358	SL264969	SL340725	SL375741	SL391435		
	SL215244	SL217470	SL218331	SL219813	SL240838	SL242375	SL271831	SL340726	SL375745	SL391464		
ERMM	SL204933	SL218357	SL235856	SL238883	SL240811	SL240869	SL240924	SL242373	SL264948	SL373321	SL375781	SL399350
	SL204939	SL218358	SL235857	SL238884	SL240812	SL240870	SL240926	SL242374	SL264959	SL373324	SL390297	SL399353
	SL204941	SL219800	SL235858	SL238889	SL240813	SL240873	SL240930	SL242393	SL264963	SL373326	SL390320	SL399354
	SL204947	SL219805	SL235860	SL238890	SL240818	SL240874	SL240934	SL242394	SL264965	SL373327	SL391436	SL404278
	SL204948	SL219818	SL235862	SL238897	SL240822	SL240875	SL240937	SL242395	SL264966	SL373328	SL391458	SL404301
	SL215231	SL233338	SL235866	SL238899	SL240823	SL240876	SL240939	SL242397	SL271832	SL373329	SL391459	SL404303
	SL215238	SL233341	SL235868	SL238900	SL240824	SL240878	SL240942	SL242398	SL271839	SL373330	SL391463	SL404329
	SL215240	SL233347	SL235869	SL238901	SL240825	SL240880	SL240943	SL242399	SL271841	SL373344	SL391482	SL405606
	SL215256	SL233348	SL235870	SL240773	SL240826	SL240881	SL240944	SL242401	SL271880	SL373345	SL391485	SL405608
	SL215259	SL233352	SL235872	SL240775	SL240828	SL240883	SL240945	SL250118	SL271908	SL373346	SL399198	SL405609
	SL215261	SL233358	SL235874	SL240776	SL240829	SL240884	SL240950	SL250119	SL278520	SL373348	SL399199	SL405629
	SL215262	SL233360	SL235876	SL240777	SL240830	SL240888	SL240951	SL250130	SL310703	SL373350	SL399202	SL405630
	SL215267	SL233370	SL235878	SL240778	SL240835	SL240890	SL240952	SL250142	SL310739	SL373351	SL399203	SL405634
	SL217463	SL233374	SL238863	SL240781	SL240837	SL240892	SL240953	SL250146	SL310740	SL373355	SL399316	SL405724
	SL217464	SL233377	SL238865	SL240784	SL240839	SL240894	SL240955	SL253595	SL316610	SL373356	SL399318	SL405726
	SL217465	SL233378	SL238866	SL240785	SL240843	SL240895	SL240956	SL253596	SL316631	SL373367	SL399319	SL405727
	SL217466	SL235834	SL238867	SL240787	SL240844	SL240896	SL240957	SL253598	SL316711	SL373369	SL399321	SL405729
	SL217467	SL235838	SL238868	SL240789	SL240845	SL240899	SL240958	SL253600	SL322916	SL373371	SL399322	
	SL217496	SL235841	SL238869	SL240792	SL240847	SL240905	SL240964	SL258025	SL322930	SL373375	SL399323	
	SL217498	SL235844	SL238870	SL240794	SL240848	SL240906	SL242323	SL258045	SL340723	SL373377	SL399339	
	SL217499	SL235845	SL238873	SL240795	SL240854	SL240907	SL242334	SL264893	SL340724	SL375746	SL399342	
	SL217504	SL235846	SL238874	SL240800	SL240855	SL240909	SL242335	SL264914	SL340739	SL375748	SL399343	
	SL217505	SL235847	SL238875	SL240801	SL240862	SL240911	SL242345	SL264938	SL340746	SL375766	SL399345	
	SL217507	SL235848	SL238877	SL240803	SL240866	SL240915	SL242359	SL264942	SL340747	SL375767	SL399346	
	SL218334	SL235852	SL238879	SL240805	SL240867	SL240919	SL242369	SL264943	SL340789	SL375768	SL399348	
	SL218345	SL235854	SL238882	SL240807	SL240868	SL240922	SL242371	SL264945	SL373320	SL375777	SL399349	
LRMM	SL204931	SL217500	SL233376	SL238878	SL240840	SL240917	SL242396	SL264913	SL316663	SL373357	SL390321	SL404328
	SL204934	SL217501	SL233379	SL238885	SL240841	SL240918	SL250107	SL264915	SL316664	SL373373	SL390345	SL404349
	SL204936	SL217502	SL233380	SL238886	SL240846	SL240921	SL250173	SL264916	SL316665	SL375744	SL391460	SL405605
	SL204943	SL218359	SL233381	SL238887	SL240851	SL240927	SL250183	SL264957	SL316666	SL375769	SL391461	SL405632
	SL204945	SL233335	SL235849	SL238888	SL240857	SL240929	SL250184	SL271838	SL316679	SL375770	SL391462	SL405633
	SL204949	SL233346	SL235859	SL238898	SL240859	SL240932	SL253572	SL271845	SL316727	SL375771	SL391480	SL405635
	SL215225	SL233349	SL235861	SL238902	SL240861	SL240935	SL253573	SL271847	SL322902	SL375772	SL391481	
	SL217476	SL233350	SL235863	SL238903	SL240863	SL240941	SL253574	SL271886	SL322904	SL375773	SL391483	
	SL217477	SL233361	SL235864	SL238904	SL240865	SL240954	SL253602	SL271926	SL322917	SL375774	SL399174	
	SL217479	SL233363	SL235867	SL240779	SL240872	SL240959	SL257980	SL278555	SL340722	SL375775	SL399200	
	SL217481	SL233364	SL235871	SL240782	SL240885	SL240961	SL258022	SL310718	SL340761	SL375776	SL399315	
	SL217482	SL233366	SL235873	SL240783	SL240886	SL240962	SL258024	SL310733	SL340762	SL375779	SL399320	
	SL217487	SL233371	SL235875	SL240788	SL240891	SL240963	SL258044	SL316611	SL340763	SL375785	SL399341	
	SL217488	SL233372	SL235877	SL240791	SL240893	SL242309	SL258047	SL316643	SL340790	SL375787	SL404302	
	SL217489	SL233373	SL235879	SL240815	SL240903	SL242327	SL258049	SL316647	SL373331	SL375788	SL404326	
	SL217494	SL233375	SL238864	SL240816	SL240910	SL242336	SL264891	SL316648	SL373347	SL390296	SL404327	

Supplementary Table 6

Supplementary Table 6. RNASeq patient ID and disease status map.

FOR REVIEWER'S USE ONLY

We are including this data for reviewers only, as it is crucial to demonstrate the quality of CD138+-enriched samples used in this manuscript. This data is part of a more detailed description and characterization study that will be published by our collaborators.

Figure 0 for Reviewer's use only shows 8 NDMM (Newly Diagnosed Multiple Myeloma) patient BM specimens that were subjected to single-cell RNA-seq in both pre-CD138-selection (pre-sort) and post-CD138-selection (post-sort). The pie chart shows the proportion of various cell types identified using the Seurat package in R, where the section of the pie chart denoted in white represents the percentage of plasma cells within the total population. We see that regardless of the initial proportion of plasma cells in the pre-sort sample, plasma cells are significantly enriched post-sort. One reasons for such high-CD138+ selection stems from our protocol that allows us to draw a 20-cc specimen, which is more than what may be allowed in other medical centers (typically, 5-cc). The higher the volume of the sample the higher the quality of CD138 enrichment, as demonstrated in **Figure 0**.

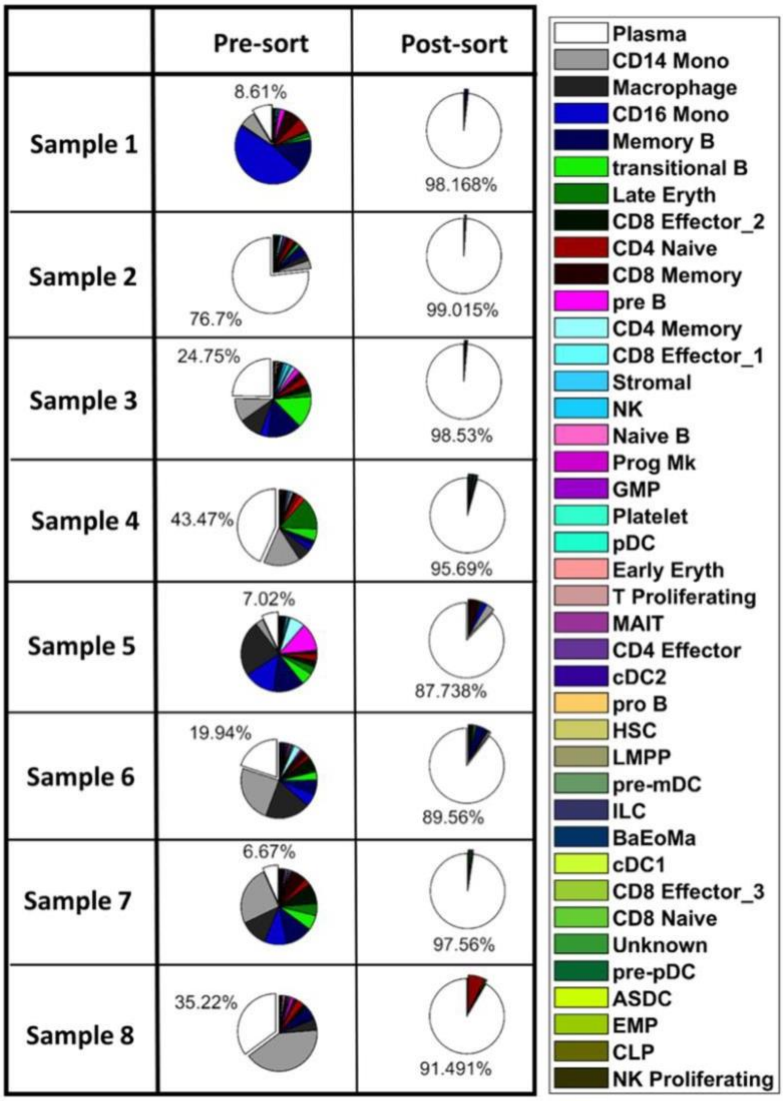

Figure 0 for Reviewer's use only | Quality of CD138+-enriched Samples Shown Using Single-cell RNA Sequencing Data Subjected to Cell Type Identification

Title: Unc-51 Like Kinase 3 (ULK3) is essential for autophagy and cell survival in multiple myeloma.

Authors: Marilena Tauro[1], Tao Li[1], Praneeth R. Sudalagunta[2], Mark Meads[1,4], Rafael Renatino Canevarolo[2], Niveditha Nerlakanti[1], Raghunandan R. Alugubelli[4], Harshani R. Lawrence[6], Steven Gunawan[5], Mohammad Ayaz[5], Pradeep Nareddy[5], Sang Young Yun[5], Gemma Shay[1], Kathy Yang[6], Timothy H. Tran[6], Ryan T. Bishop[1], Mostafa M. Nasr[1,3], Nicholas N.J. Lawrence[5], Ernst Schonbrunn[5], John L. Cleveland[1], Ariosto S. Silva[3], Kenneth H. Shain[1,4] and Conor C. Lynch[1*]

SUPPLEMENTAL METHODS

Study design

Ethical Considerations: The utilization of $CD19^+$ B cells from donors at H Lee Moffitt Cancer Center was conducted with explicit informed consent and was approved by the Institutional Review Board (IRB) under protocol #: Pro00021733. Similarly, $CD138^+$ primary cells were sourced from patients enrolled in clinical trials/protocols (MCC17814, MCC14745, MCC14690, and MCC18608) at H. Lee Moffitt Cancer Center and Research Institute. These protocols were conducted in accordance with ethical standards outlined in the Declaration of Helsinki, International Ethical Guidelines for Biomedical Research Involving Human Subjects (CIOMS), Belmont Report, and U.S. Common Rule, ensuring adherence to patient privacy and ethical treatment.

For RNAseq data of multiple myeloma patients, the handling of patient data adhered to rigorous privacy measures as medical records were deidentified. Only clinically relevant information was scrutinized, including (A) details of administered treatments (therapeutic agents, doses, and schedule) preceding the biopsy, (B) cytogenetic information, and (C) results from serum and urine electrophoresis. This approach ensures both the ethical treatment of participants and the meticulous examination of pertinent clinical details in compliance with established ethical norms and regulations.

Animal Experimentation: All animal experiments were performed with approval from the Moffitt/University of South Florida Institutional Animal Care and Use Committee (Protocol #10955R, Title: Role of ULK3 in Sensitive and Refractory Multiple Myeloma). For *in vivo* validation of MA9 efficacy (**Figure 4**), we initially included n = 10 mice per treatment group:

suboptimal tail vein injection resulted in exclusion criteria. Randomization based on sex, weight, and initial tumor burden was conducted, followed by treatment with either vehicle control (n = 6), single agents MA9 (n = 9) and JQ1 (n = 9), CQ (n = 10), or the combination of JQ1 and CQ (n = Tumor progression was tracked via bioluminescence: endpoint was paralysis or >20% weight loss. Experiments were blinded. For the NTC CTRL vs ULK3 KO U266 in vivo model, mice (n=10/ group) received 1×10^6 cells via tail vein (**Supplementary Fig. 7**).

For MA9 therapy efficacy, mice were treated with vehicle (n=10) or MA9 (10 mg/kg/day, subcutaneously, M-F from day 20; n=10) (**Supplementary Fig. 7E, F**). Survival and toxicity (weight, clinical signs) was monitored.

Cell culture

Luciferase-labeled MM cells 5TGM1-Luc (RRID: CVCL_VI66) and U266-Luc (RRID: CVCL_0566) were obtained from University of Texas, Health Science Center at San Antonio, TX (2012) and University of Virginia, VA (2014), respectively. MM1.S cell line was obtained in 2015 from ATCC (RRID: CVCL_8792). Isogenic PI-sensitive and resistant pairs U266 and PSR [1], RPMI8226 (RRID: CVCL_0014) and B25 [2] were obtained from Dr. Steven Grant's laboratory (VCU-Massey) and ANBL-6 (RRID: CVCL5425) and V10-R [3] pairs were obtained from Dr. Kenneth Shain's laboratory (Moffitt Cancer Center). PI-resistant cell lines were maintained through bi-weekly dosing with PI (bortezomib, 30 nM). Human MM.1S, U266, RPMI 8226 MM cells (all from the ATCC, USA) were routinely cultured at 37 °C in Roswell Park Memorial Institute (RPMI) 1640 medium supplemented with 10% heat-inactivated FBS (PEAK, CAT# PK-FBS1) and penicillin/streptomycin (hereafter, complete medium).

Cells were not used for five days following PI-treatment. All myeloma cell lines were maintained below $1x10^6$ cells/ml in complete medium and used within 20 passages. IL-6 dependent cell lines ANBL-6 and V10-R are cultured in complete medium and 1ng/ml of IL-6 (R&D systems, HZ-1019). Isolation and culture methods for mesenchymal stem cells (MSCs) were adapted from previously published protocols [4]. Briefly, hind limbs were collected from tumor-I 4–6-week-old male C57/BL6 *Rag2*$^{-/-}$ mice in sterile PBS. Following removal of muscle tissue, epiphyses were removed, and bone marrow flushed three times with sterile PBS to deplete the hematopoietic cells. Flushed bones were then cut into 1-3 mm chips, digested with 1 mg/mL collagenase II (Invitrogen) in α-MEM with 15% FBS, and shaken at 150 RPM for 1 h at 37 °C.

Digested bone fragments were grown in 6-well tissue culture plates in α-MEM with 15% FCS. Medium was changed every 3 days [5].

For murine osteoclast culture, whole bone marrow was flushed from the tibia of 6–8-week-old male or female C57BL/6 *Rag2*$^{-/-}$ mice and cultured in the presence of α-MEM and 25ng/ml macrophage colony stimulating factor (M-CSF CAT# 315-02, Peprotech) for 3 days. Non-adherent bone marrow cells were maintained in osteoclastogenic medium (100 ng/ml RANKL CAT# 47187000, OYC Americas and 20 ng/ml M-CSF) for 7 days [6].

Cells used in these studies routinely tested negative for mycoplasma by PCR (Polymerase Chain Reaction) (Bulldog Bio, CAT # 25233), and were additionally authenticated against ATCC, DSMZ or ExPASy STR profiles.

WM1366 cell line stably transfected with m-cherry-GFP-LC3 was a kind gift of Dr. Keiran Smalley (H Lee Moffitt Cancer Center) and was generated and selected as previously described [7].

RNA Sequencing (RNA-Seq)

Nucleic Acid Extraction: RNA extraction from frozen tissue was performed using the Qiagen RNeasy Plus Mini Kit, resulting in an average insert size of 216 bp.

RNA Sequencing: The Illumina TruSeq RNA Exome protocol was employed, involving single library hybridization, cDNA synthesis, library preparation, and sequencing with either 100 or 150 bp paired end reads, achieving a total coverage of 100 million reads or 50 million paired reads. The RNA-Seq data was analyzed using the Tumor Pipeline, following the workflow below with the GRCh38/hg38 human genome reference and GenCode build version 32.

Adapter Trimming: Adapter sequences were removed from the raw sequencing FASTQ files. This process involved k-mer matching for adapter trimming, along with quality trimming, contaminant removal, sequence masking, GC content filtering, length filtering, and entropy filtering. The processed FASTQ files were then used for reading alignment.

Read Alignment: The trimmed FASTQ files were aligned to the human genome reference (GRCh38/hg38) and the Gencode genome annotation v32 using the STAR aligner. The STAR aligner produced several output files for subsequent gene fusion prediction and gene expression analysis.

RNA Expression: Expression levels were calculated based on mapped reads, with values reported as Fragments Per Kilobase of transcript per Million mapped reads (FPKM) and Transcripts Per Million mapped reads (TPM), both at the transcript and gene levels, using transcriptome alignments from STAR.

Gene Expression Data Download and Normalization: Gene expression data were retrieved from DNAnexus files containing FPKM and TPM values for 59,368 records. Of these, 19,933 were protein-coding genes, which were further analyzed, while the rest were excluded. For each gene/sample, log2(FPKM + 10^-3) values were calculated, and genes with identical values for the first and third quartiles (indicating expression in less than 25% of samples) were excluded. The remaining 16,738 genes were z-normalized across all samples using MATLAB's normalization function.

Cloning, ULK3 protein expression and purification

The gene encoding human *ULK3* (amino acid 8-274) was synthesized and subcloned into pET28a (+)-TEV vector (GeneScript) and co-expressed with lambda phosphatase as N-terminal His6 and GFP fusion protein in E. coli strain BL21(DE3). Cells were harvested 20 hours post induction by 0.1 mM IPTG and resuspended in lysis buffer containing 50 mM HEPES, pH 7.4, 500 mM NaCl, 20 mM imidazole, 0.5 mM TCEP (Tris(2-carboxyethyl) phosphine), 10 µM MnCl2, 5% glycerol, 0.1% Triton X-100, 1 tablet of EDTA free protease inhibitor cocktail/50 mL buffer. The supernatant of the cell lysate was applied to Ni-NTA Superflow resin (Qiagen), and the fusion protein was eluted with buffer composed of 50 mM HEPES, pH 7.4, 500 mM NaCl, 300 mM imidazole, 0.5 mM TCEP, 5% glycerol by a liner gradient method. Fractions containing the tagged protein were combined and desalted by Sephadex G-25 packed column (GE Healthcare Life Science), followed by His-GFP tag removal by tobacco etch virus protease with gentle stirring at 4°C overnight. The cleaved mixture was passed over the Ni-NTA Superflow resin again and the untagged protein was further purified via size exclusion chromatography using Superdex 75 26/60 column (GE Healthcare Life Science) in 25 mM HEPES, pH 7.4, 150 mM NaCl, 0.5 mM TCEP. The purified protein was analyzed by sodium dodecyl sulphate-polyacrylamide gel and the concentration was determined by NanoDrop 2000c Spectrophotometer. The protein was flash-frozen in liquid nitrogen and stored at -80°C.

Immunoprecipitation & Immunoblotting

LC-MS/MS Sample preparation: Bead-bound proteins were resuspended in 20 µL of SDS-PAGE MOPS running buffer (BioRad CAT# 1610788), 10 µL of loading buffer (BioRad CAT# 1610791), and 2 µL of reducing agent (BioRad CAT# 1610792), then boiled and denatured at 95 °C for 5 minutes. Samples were cooled and loaded onto a 10% Bis-Tris Criterion XT Precast Gel (BioRad CAT# 345-0111). SDS-PAGE was performed at 125 V for 2 hours. The gel bands were then stained with Instant Blue Coomassie stain (Abcam CAT# ab119211), imaged, and cut. An in-gel digestion was performed with 2 mM TCEP reduction and 20 mM IAA alkylation followed by digestion with 200 ng of trypsin overnight at 37 °C. Another aliquot of 200 ng trypsin was added the next day for an additional 2-hour digest. Peptides were extracted from the gel pieces using 50% acetonitrile, 0.1% trifluoroacetic acid, then dried down in a vacuum centrifuge. Peptides were resuspended in 200 µL of 0.1% trifluoroacetic acid for C18 desalting using a SOLAu plate (Thermo CAT# 60209-001). Eluted, desalted peptides were assayed via a peptide concentration assay (Pierce CAT# 23275). 100 ng of peptide were vacuumed centrifuged to dryness and resuspended in 30 µL of 0.1% trifluoroacetic acid, then loaded on EvoSep EvoTip Pure tips in preparation for LC-MS/MS analysis.

Analysis: A nanoflow ultra-high performance liquid chromatograph and nano electrospray orbitrap mass spectrometer (EvoSep and Q-Exactive plus) were used for LC-MS/MS. The sample was loaded onto a EvoTip pure (EV2013). Trapped peptides were eluted onto the analytical column (EV1106, 15 cm length x 150 µm ID, 1.9 µm particle size). A factory default extended gradient (88-minute) was used with solvent A (water + 0.1% formic acid) and solvent B (acetonitrile + 0.1% formic acid). The spray voltage was 1900 V. Capillary temperature was 275 °C. S lens RF level was set at 50. Data-dependent acquisition was performed using Top16 precursors. The resolution for MS (Mass Spectrometry) and MS/MS were set at 70,000 and 17,500 respectively. Dynamic exclusion was 15 seconds for previously sampled peaks.

Data Analysis: Raw files were loaded into Proteome Discoverer 3.0 for database searching with Mascot and Sequest. Enzyme was set to Trypsin, allowed missed cleavage of 2, precursor mass tolerance of 10 ppm and fragment mass tolerance of 0.05 Da, SwissProt Human 2023_03 protein database, with variable modifications of carbamidomethylation of cysteine and oxidation of methionine. Scaffold 5.0 and Skyline version 23.1 were used to visualize the data.

Immunoblotting: After the corresponding treatments, pelleted cells were lysed by RIPA buffer supplemented with proteinase/phosphatase inhibitor cocktail (Cell Signaling Technology CAT# 5872S). Protein concentration was quantified by Thermo Scientific™ Pierce™ BCA™ Protein Assay (Fisher Scientific CAT# PI23225). 30 µg protein was loaded to 10%-15% gel and ran at 100V for 2 hours. Later protein was transferred to PVDF membrane and blocked with 5% skimmed milk in TBST (10 mM Tris/HCl pH8.0, 0.12M NaCl, 0.1% Tween 20) containing 5% BSA [8] for 1 hour.

Primary antibodies were diluted at 1:1000 ratio in skimmed milk and incubated with the membrane at 4°C overnight. Membranes were washed 3 times,10 minutes with TBS. Then incubated with appropriate secondary antibodies at 1:1000 ratio in skimmed milk or 2% BSA. The membranes were washed again 3 times for 10 minutes with TBST. ECL reagents were added to the membrane and blot was developed by LICOR Imaging Studio.

All immunoblotting assays were performed at least in three replicates and representative images are shown. Commercially available antibodies and their dilutions are listed in **Supplementary Table 5**. Immunoblotting images were acquired and quantified using LICOR Image Studio Quant software.

Immunofluorescence staining

For TMA analyses, slides were deparaffinized and then underwent antigen retrieval by immersion in Antigen Retrieval Citra Plus solution (pH-6.2) (BioGenex, SKU: HK080-9K) for 45 minutes in steamer conditions. After cooling down, slides were blocked in PBS with 10% goat serum for 1 hour at room temperature (RT), then incubated for 1 hour with primary antibodies (FITC-conjugated kappa/lambda light chains, Dako Agilent F0198/ F0199, 1:500 dilution, Alexa Fluor647 anti-ULK3 [EPR4888], CAT# ab310161, 1:50). After 3 x 10 min washes after incubation, 0.1 µg/mL DAPI (CAT# 28718-90-3 Sigma Aldrich) with Vector TrueVIEW (Vector Labs) autofluorescence quenching reagent was used prior to mounting the slides. Confocal immunofluorescent images of each TMA core were acquired with a Leica SP8 system with 63x objective (Leica). 405, 488, and 638 laser lines were used to excite the fluorophores and emissions were tuned to detect fluorophore specific wavelengths. A fourth channel was added using the 552-laser line to detect autofluorescence in the orange to red part of the spectrum. The autofluorescence channel was used to observe platelets and red blood cells (RBCs) within the tissue.

Quantification: The fluorescence LIF image file was imported into QuPath 0.4.3 where TMA core images were analyzed. First, tissue areas were manually annotated to ignore bone tissue, debris, and regions with a heavy number of platelets and RBCs. Corresponding hematoxylin and eosin (H&E) stained cores were used to guide this process. Second, the cell detection algorithm was applied to these annotated regions using the DAPI channel to segment every cell into an individual object. A threshold classifier was used with the autofluorescence channel to separate the platelets and RBCs from other cell types. Third, a second threshold classifier was used with the CD138 (green fluorescence) channel to identify plasma cells within the previously identified cell populations. Finally, a pixel-based classification was used to identify ULK3 staining area within each cell using a minimum intensity threshold of 50 (on a scale 0-255). Data for each cell including area and staining intensity measurements were exported to Microsoft Excel for data analysis.

For *in vitro* immunofluorescent detection of ULK3 in MM cell lines, cells were grown on glass coverslips, fixed with ice cold methanol for 10 minutes and blocked in 10% Normal Goat Serum for 1 hour. Cells were then incubated for 1 hour with primary antibody (Recombinant Anti-ULK3 antibody [EPR4888] CAT# ab124947), washed and incubated with secondary antibodies for 1 hour. After washing, cells were mounted with 10 µl of mounting media (VECTASHIELD antifade mounting medium with DAPI, Vector Laboratories CAT# H-1200) and imaged by confocal microscopy. Antibodies used are listed in **Supplementary Table 5**.

For *ex vivo* immunofluorescent detection of proteins in xenograft tumors (isolated from U266Luc model), formalin fixed, paraffin embedded tumors were cut at 5 microns sections. Deparaffinized tissue slices underwent rehydration and antigen retrieval by immersion in Antigen Retrieval Citra Plus solution (pH-6.2) (BioGenex, SKU: HK080-9K) for 45 minutes in steamer conditions. After cooling down, slides were blocked in PBS with 10% goat serum and stained for ULK3 (Alexa Fluor® 647 Anti-ULK3 antibody [EPR4888] (CAS# ab310161)) and MM cells (anti-human k**λ** light chain Dako F0198, F0199 in green) colocalization. Confocal immunofluorescent images were acquired with Leica TCS SP8 system with 40x objective (Leica) and quantified as number of tumor cells positive for ULK3/ total number of cells. Mean of each antibody staining was used to determine threshold of positivity.

For ki67 and cleaved caspase staining, slides were hydrated in xylene (10 minutes x 2), 100% ethanol (2 minutes x 2), 90% ethanol (2 minutes x 2), 70% ethanol (2 minutes x 2), 50% ethanol

(2 minutes x 2), and rinsed in water for 3 minutes. Tissues were incubated in gently boiling pressure cooker buffer (Tris-EDTA Buffer, pH=9) for 4 minutes/high for antigen retrieval step. Slides were cooled down to RT and blocked with 10% Normal Goat Serum in PBS for 1 h. Primary antibody Ki67 (rabbit, 1:1000, CAT# 91295) or CC3 (rabbit, 1:1000, CAT# 9664L) and human MM cells (hMITO, mouse, 1:500, CAT# MAB1273) were diluted in 10% Normal Goat Serum in PBS and incubated with the slides at RT for 1 hour. Slides were washed with TBS for 5 minutes 3 times. Alexa Fluor Plus 647 goat anti-rabbit (1:1000, Invitrogen CAT# A32733), and Alexa Fluor Plus 488 goat anti-mouse (1:1000, Invitrogen CAT# A32723) were used as secondary antibodies for 1 hour at room temperature. After washing, cells were mounted with 10 µl of mounting media (VECTASHIELD antifade mounting medium with DAPI, Vector Laboratories CAT# H-1200) and imaged by confocal microscopy.

Confocal microscopy: Tissue slides were imaged with a Leica SP8 confocal microscope (Leica Microsystems GmbH, Wetzlar, Germany). Samples were excited with 405, 488, and 638 laser lines and emissions were tuned to appropriate wavelengths for each dye. Images were captured through a 40X/1.3NA objective lens with and without a 2X zoom using sequential acquisition. All system settings remained consistent for all samples within the experiment. Images were processed for maximum projections and exported in TIF format with LAS X software version 3.1.5 (Leica Microsystems GmbH, Wetzlar, Germany).

The TIF images were imported into Definiens Tissue Studio version 4.7 (Definiens AG, Munich, Germany) for image analysis. The software was used to segment individual nuclei using a nucleus detection algorithm applied to the Hoechst channel. Within each nucleus the co-expression detection algorithm was used to detect areas containing both green fluorescence and red fluorescence and areas without co-expression. Identical intensity thresholds were used for each image within an experiment. Nuclei count and area data for each nucleus was exported to excel for further data analysis.

Genetic manipulation of ULK3 expression

CRISPR-CAS9: DNA electroporation was performed with Neon® Transfection System (Thermo Fisher, MPK5000) and Neon® Transfection System 10 µl Kit (CAT# MPK1096), as per manufacturer's guidelines [9]. For the electroporation with the Neon System, cells were grown to

80% confluence. Cells were pelleted again and re-suspended in Resuspension Buffer R (Neon 10 µl kit Invitrogen) to a final concentration of 1×10^7 cells/ml. 1×10^6 cells were then transferred to a sterile 1.5 ml microcentrifuge tube (Sarstedt), brought to 10 µl final volume of cell suspension with Buffer R, and mixed with Alt-R CRISPR-Cas9 crRNA: Hs.Cas9.ULK3.1.AA (position 74840611, CGCACAACACATGTCCCCGT); Hs.Cas9.ULK3.1.AB (position 74839605, AGGACTTTTTTG CGCACCCC), (Integrated DNA Technologies). Alt-R CRISPR-Cas9 Negative Control RNA #1 (CAT# 1079138) was used in NTC control._Cells were counted, electroporated and seeded in a 24-well plate with 0.5 ml of pre-warmed supplemented RPMI without antibiotics.

Measurement of autophagic activity - CytoID Assay

The Cyto-ID Green autophagy dye served as a selective marker of autolysosomes and early autophagic compartments. Cells were washed and treated with 1 µM tested compound or vehicle control for 6h under standard tissue culture conditions. Upon completion of treatment period, cells were washed with PBS and seeded at 10% confluency in FluoroDish (WPI FD35-100, glass bottom for confocal microscopy) or $1x10^5$ cells/100ul (flow cytometry). Microscopy: for every 1 mL of 1X Assay Buffer or complete cell growth medium, 2 µL of CYTO-ID® Green Detection Reagent and 1 µL of Hoechst 33342 Nuclear Stain were added and supplemented with 1% FBS.

Flow cytometry: The assay buffer containing 1% FBS, 1 µl of Cyto-ID Green Detection Reagent in 1 ml total volume and 100ng/ml DAPI Live/dead staining was added. Suspension cells were mixed well and incubated under standard tissue culture conditions in the dark. 30 minutes later, cells were washed and assessed by flow cytometry. Stained controls and samples were analyzed using BD Biosciences LSRII flow cytometer or BD FACS Canto. Appropriate compensation and fluorescence-minus-one (FMO) controls were generated in parallel. The median fluorescence intensity (MFI) and percentage of Cyto-ID Green-expressing MM singlet live (DAPI negative) human MM cells in FCS Express 7. Gating strategy is reported in **Supplementary Fig. 8**.

Confocal Microscopy: cells stained with Hoechst 33342 Nuclear Stain and Cyto-ID were imaged with a Leica SP8 confocal microscope (Leica Microsystems GmbH, Wetzlar, Germany). Samples were excited with 405 and 488 laser lines and emissions were tuned to appropriate

wavelengths for each dye. Images were captured through a 40X/1.3NA objective lens with a 2X zoom using sequential acquisition on two PMT detectors. All system settings remained consistent for all samples within the experiment. Images were exported in TIF format with LAS X software version 3.1.5 (Leica Microsystems GmbH, Wetzlar, Germany).

Analysis: the TIF images were imported into Definiens Tissue Studio version 4.7 (Definiens AG, Munich, Germany) for image analysis. The software was used to segment individual nuclei using a nucleus detection algorithm applied to the Hoechst channel. Within each nucleus the Spot detection algorithm was used to detect green fluorescence spots with identical intensity and size thresholds for each image within an experiment. Spot count and area data for each nucleus was exported to excel for further data analysis.

Microsome stability assay

The assay was conducted by the Exploratory Pharmacology Core at the Sanford Burnham Prebys Medical Discovery Institute in Orlando, Florida, following established procedures as previously reported [34]. Compounds, at a final concentration of 1 μM, were incubated with hepatic microsomes (1 mg/mL) for a duration of 1 hour, as detailed in the referenced protocol [34]. The quantification of the remaining compound was determined through High-Performance Liquid Chromatography (HPLC).

ULK3-MA9060 crystallization and structure determination

The frozen protein sample was thawed, concentrated to ~12 mg/ml, mixed with ~500 μM compound MA9-060 and incubated for at least 1 hour. Crystallization screenings were carried out using the sitting-drop vapor diffusion method. Initial crystal hits were obtained using a reservoir solution containing 100 mM amino acid cocktail (L-Na-Glutamate; Alanine (racemic); Glycine; Lysine HCl (racemic); Serine (racemic)), 100 mM buffer 1 cocktail (Imidazole; Sodium Cacodylate; MES (acid); Bis-Tris), and 30% glycerol and PEG 4000 mix. The crystals were further optimized and flash-frozen in liquid nitrogen directly without additional cryo-protectant. Xray diffraction data were collected at 100 K, on beamline 19-ID of NSLS-II synchrotron at Brookhaven National Laboratory. The data was integrated and scaled using XDS [12]. Structure solution was obtained by molecular replacement using Phaser as implemented in the Phenix programs suite, using a search model PDB:6FDY published previously [13]. Iterative rounds of model building

and refinement were performed with COOT [14] and Phenix.Refine [13] to obtain a clear electron density map (1Fo-Fc) for compound MA9-060, which was built into the structure near the end of iterative cycles of model building and refinement. Crystal parameters, data collection statistics and refinement statistics are summarized in **Supplementary Fig. 4D** and deposited in the protein databank with PDB code 8V1M.

Kinase profiling assay

The compounds under investigation were tested by Reaction Biology Corporation [15] against four specific kinases in this study (JAK2, ULK1, ULK2, ULK3). Each compound was provided as a 10 mM stock solution. To facilitate dissolution, the compounds were subjected to gentle heating. Subsequent testing involved the evaluation of the compounds using a 10-dose IC_{50} mode, employing a 3-fold serial dilution starting at 10 µM. Additionally, the control compound, Staurosporine, underwent testing in a 10-dose IC_{50} mode with a 4-fold serial dilution starting at 20 µM. Throughout these experiments, reactions were conducted using a standardized concentration of 10 µM ATP.

The data collected during these assays encompass raw data, % enzyme activity (relative to DMSO controls), and curve fits. Notably, curve fits were specifically performed in cases where the enzyme activities at the highest concentration of compounds were less than 65%. The IC_{50} summary, detailing the inhibitory concentrations of the compounds against the respective kinases, is presented in **Supplementary Fig. 4A**.

Bromodomain profiling assay

Briefly, four compounds (SG3-014, MA9-060, JQ1, CQ; stock concentration of 10 mM) were evaluated against Bromodomain 1 by Reaction Biology Corporation.

The compounds were subjected to testing using a 10-dose IC50 singlet approach, involving a 3-fold serial dilution starting at 10 µM, specifically against BRD4-1. Additionally, as a benchmark, the control compound JQ-1 underwent testing in a 10-dose IC_{50} mode, employing a 3-fold serial dilution starting at 10 µM.

For ligand and detection purposes, the H4/4Ac ligand, representing the Histone H4 peptide (1-21) with acetylation at lysine residues K5, K8, K12, and K16, and tagged with Biotin, was utilized.

Detection was carried out through the AlphaScreen Binding assay with excitation/emission wavelengths set at 680/520-620 nm, respectively, using the Enspire platform.
The data acquisition included raw data, represented as signal-background*, % Binding (relative to DMSO controls), and curve fits. Notably, curve fits were performed where the % Binding at the highest concentration of compounds exhibited significant variation from DMSO controls. The IC_{50} summary, detailing the inhibitory concentrations of the compounds against the respective kinases, is presented in **Supplementary Fig. 4A**.

mCherry-GFP-LC3 fluorescence intensity quantification and imaging

WM1366 mCherry-GFP-LC3 cells were obtained as a kind gift of Dr. Keiran Smalley's collaboration with The Wistar Institute and produced as previously described [7].
For cell imaging, WM1366 mCherry-GFP-LC3 cells were seeded onto glass coverslips. Next day, medium was refreshed with RPMI containing 10% fetal bovine serum, and treated with vehicle control, SG3-014, MA9-060, JQ1, CQ (0.1 μM). After 6 hours, cells were fixed and permeabilized with ice cold MeOH.
Imaging was performed with an inverted Leica SP8 confocal microscope equipped with an 40X/1.3NA oil lens objective. Cells were mounted in a 35 mm FluoroDish Cell Culture Dish (WPI, Sarasota, FL), and z-stacks were acquired at a speed of 400 Hz approximately 30 min post-treatment and every 2 hours thereafter. Two to three fields were acquired per sample. Field size was 388x388 μm in x and y, and 9 μm in z, with a voxel size of 0.4x0.4x1 μm. Excitation and emission wavelengths were at 488 and 552 nm, and 500-538 nm and 587-656 nm respectively. Z-stacks were then max projected for visualization. (From Moffitt Microscopy Shared Resources). Analysis of images was performed with Zen software (Zeiss) or ImageJ software (NIH, National Institutes of Health).

***In vitro* osteoclast differentiation assays**

Whole bone marrow was flushed from the tibia of 6–8-week-old male or female C57BL/6 *Rag2*$^{-/-}$ mice and cultured in the presence of α-MEM and 25 ng/ml macrophage colony stimulating factor (M-CSF CAT# 315-02, Peprotech) for 3 days. Non adherent bone marrow cells were maintained in osteoclastogenic medium (100 ng/ml RANKL CAT# 47187000, OYC Americas and 20 ng/ml M-CSF) for 7 days [6, 16]. SG3-014 or JQ1 were added at varying concentrations (10 to 50 nM)

for 24 hours. Vehicle control wells were treated with 0.5% DMSO that reflected the final concentration of the vehicle in the 50 nM drug treated groups. Subsequently, osteoclasts were fixed with 4% paraformaldehyde, and TRAcP stained as previously described. TRAcP positive multinucleated (^{3}3 nuclei) osteoclasts were counted using ImageJ [17].

Bone sectioning and histology

The harvested tibiae were fixed with 10% neutral formalin for 24 h and decalcified with 10% EDTA for 4 weeks, prior to dehydrating by a graded alcohol series, and embedding in paraffin. Tibia tissue sections (5 µm) were stained with hematoxylin and eosin (H&E) using well-established and standardized protocols as previously outlined [16].

Osteoclast TRAcP staining

For TRAcP staining and osteoclast measurements, slides were deparaffinized and rehydrated to water then incubated in Basic Stock Incubation Medium (112mM anhydrous sodium acetate, 49mM dibasic dehydrate sodium tartrate, 0.28% glacial acetic acid) containing 1% Naphthol-Phosphate substrate (2% Napthol AS-BI Phosphate in 2-Ethoxyethanol) for 1 hr at 37°C. Slides were then transferred to Basic Stock Incubation Medium containing 250 µL Pararosaniline dye (5% pararosaniline dye in 2N HCl) and 250 µL of sodium nitrite solution (4% sodium nitrite in distilled water) at 37°C and monitored for development of red stained osteoclasts. After developing, the slides were rinsed in distilled water and counterstained with Hematoxylin, blued, and aqueously mounted. The number of multi-nucleated, TRAcP positive osteoclasts were quantified from multiple tissue sections.

***Ex vivo* X-rays bone imaging**

Radiographic images were captured using the Faxitron X-ray Corp system, employing an energy setting of 35 kVp and an exposure time of 8 ms. The spatial resolution was 10 lp/mm (48 µm). The tumor area (TuA) was calculated as a function of the total tissue area (TotA) of the tibial medullary canal by using ImageJ software [18].

***Ex vivo* drug sensitivity characterization**

An *ex vivo* assay was used to quantify the chemosensitivity of primary MM cells. Fresh BM

aspirate cells were enriched for CD138+ expression using Miltenyi (Bergisch Gladbach, Germany) 130-051-301 antibody-conjugated magnetic beads. MM cells (CD138+) were seeded in Corning Cell BIND 384 well plates (Corning, NY) with collagen I and previously established human-derived stroma, containing approximately 4000 MM cells and 1000 stromal cells. Each well was filled with 80µL of RPMI 1640 media supplemented with fetal bovine serum (FBS, heat inactivated), penicillin/streptomycin, and patient-derived plasma (10%, freshly obtained from patient's own aspirate, filtered) and left overnight for adhesion of stroma. The next day, drugs were added using a robotic plate handler so that every drug/combination was tested at 5 (fixed concentration ratio, for combinations) concentrations (1:3 serial dilution) in two replicates. Negative controls (supplemented growth media with and without the vehicle control dimethyl sulfoxide [DMSO]) were included, as well as positive controls for each drug (cell line MM1.S at highest drug concentration). Plates were placed in a motorized stage microscope (EVOS Auto FL, Life Technologies, Carlsbad, CA) equipped with an incubator and maintained at 5% CO_2 and 37 °C. Each well was imaged every 30 minutes for a total duration of up to 6 days.

Digital image analysis for ex vivo drug using EMMA. Digital image analysis computes the percent viability of MM cells for each time point and experimental condition (drug and concentration) [19]. This algorithm computes differences in sequential images and identifies live cells with continuous membrane deformations resulting from their interaction with the surrounding extracellular matrix. These interactions cease upon cell death. By applying this operation to all 288 images acquired for each well, we quantified non-destructively, and without the need to separate the stroma and myeloma, the effect of drugs as a function of concentration and exposure time. For each patient-drug, we have a dose-time-response surface, which is abstracted into AUC (Area Under the Curve) (**Supplementary Tables 2**), which is an area/integral measure of *ex vivo* response to therapy computed by taking an average of all *ex vivo* responses across all time (first 96 hours) and concentration.

Ex vivo Combination Effect analysis: Synergy was determined by using the method described by Sudalagunta et al [20], in which percent live cells across time and 5 serially diluted concentrations (1:3) when treated with each drug are used to compute additive response using the Bliss Independence Model. The additive response serves as a reference to determine the extent of

synergy observed in each patient sample by comparing it with the percent live cells measured when treated with the combination (at a fixed ratio of the 2 constituent single agents). Two metrics of drug sensitivity were used, area under the curve (**Supplementary Table 2**) and median lethal dose (**Supplementary Table 3**), in which the additive area under the curve/median lethal dose are compared with that of the combination to quantify the synergy seen in each patient sample. Due to the potency of the MA9 as single agent, we expressed data as positive and negative contribution to the effect of single agent PI carfilzomib (CFZ), by calculating the Combo Benefit = (AUC CFZ+MA9) – (AUC CFZ).

This *ex vivo* drug sensitivity characterization and combination effect framework using MM patient samples was developed and pioneered at H. Lee Moffitt Cancer Center and Research Institute, which resulted in several significant contributions in the field of hematological malignancies over the past five years [21-25].

Flow cytometry quantification of ULK3

U266, MM1.S or RPMI8226 MM cells were stained at 1x106 cells/ml in PBS with Zombie NIR (1:250; Biolegend cat# 423106) for 30 minutes. Cells were washed thrice in PBS, followed by fixation and permeabilization steps with Cyto-Fast™ Fix/Perm Buffer Set (Biolegend; Cat# 426803) According to manufacturer's instructions. Cells were then stained with Alexa Fluor® 647 Anti ULK3 antibody [EPR4888] (Abcam; Cat# ab310161) at a dilution of 1:200 for 30 minutes before washing and acquisition. Stained controls and samples were acquired and analyzed using BD Biosciences LSRII flow cytometer Appropriate compensation and fluorescence-minus-one (FMO) Controls were generated in parallel and analyzed in FCS Express 7 (**Supplementary Figure 1H-I**).

Statistical analysis

For the statistical analysis of independent samples, paired and unpaired Student t-tests and the Wilcoxon test were used. Differences were considered significant if $p< 0.05$ (*). Increasing levels of significance are denoted by asterisks (** $p<0.01$, *** $p<0.001$ **** $p<0.0001$ or n.s., not significant).

For comparisons involving three or more groups, one-way analysis of variance (ANOVA) with applied post hoc Bonferroni correction for multiple comparison.

For bioluminescent imaging quantification, Two-way MIXED ANOVA was performed using IBM SPSS. Pairwise comparison (Bonferroni test) has been performed upon significance of group-time interaction. Non-parametric comparisons of two or more independent samples, with equal or different sample sizes, utilized Kruskal–Wallis's test by ranks and two-sided Wilcoxon rank-sum tests. Survival analyses employed Kaplan-Meier and Log Rank Mantel Cox tests, utilizing the R and IBM SPSS (preclinical model) package survival version, and the results were plotted using Prism GraphPad version 9.

Data exploration (outliers, normality of distribution and homogeneity of variance), correlation tests, and down sampling analysis were conducted using IBM SPSS software.

References for supplemental Materials

[1] S. Chen *et al.*, "CDK inhibitors upregulate BH3-only proteins to sensitize human myeloma cells to BH3 mimetic therapies," *Cancer Res,* vol. 72, no. 16, pp. 4225-37, Aug 15 2012, doi: 10.1158/0008-5472.CAN-12-1118.

[2] S. Chen *et al.*, "A Bim-targeting strategy overcomes adaptive bortezomib resistance in myeloma through a novel link between autophagy and apoptosis," *Blood,* vol. 124, no. 17, pp. 2687-97, Oct 23 2014, doi: 10.1182/blood-2014-03-564534.

[3] J. Zhuang *et al.*, "Ubiquitin-activating enzyme inhibition induces an unfolded protein response and overcomes drug resistance in myeloma," *Blood,* vol. 133, no. 14, pp. 1572-1584, Apr 4 2019, doi: 10.1182/blood-2018-06-859686.

[4] W. Zhu *et al.*, "Mesenchymal stem cells derived from bone marrow favor tumor cell growth in vivo," *Exp Mol Pathol,* vol. 80, no. 3, pp. 267-74, Jun 2006, doi: 10.1016/j.yexmp.2005.07.004.

[5] J. J. McGuire *et al.*, "Mesenchymal stem cell-derived interleukin-28 drives the selection of apoptosis resistant bone metastatic prostate cancer," *Nat Commun,* vol. 12, no. 1, p. 723, Feb 1 2021, doi: 10.1038/s41467-021-20962-6.

[6] G. Shay *et al.*, "Selective inhibition of matrix metalloproteinase-2 in the multiple myeloma-bone microenvironment," *Oncotarget,* vol. 8, no. 26, pp. 41827-41840, Jun 27 2017, doi: 10.18632/oncotarget.18103.

[7] A. A. Kraya *et al.*, "Identification of secreted proteins that reflect autophagy dynamics within tumor cells," *Autophagy,* vol. 11, no. 1, pp. 60-74, 2015, doi: 10.4161/15548627.2014.984273.

[8] T. Mahmood and P. C. Yang, "Western blot: technique, theory, and trouble shooting," *N Am J Med Sci,* vol. 4, no. 9, pp. 429-34, Sep 2012, doi: 10.4103/1947-2714.100998.

[9] M. Beyer and O. H. Kramer, "RNA interference protocol to silence oncogenic drivers in leukemia cell lines," *STAR Protoc,* vol. 3, no. 3, p. 101512, Sep 16 2022, doi: 10.1016/j.xpro.2022.101512.

[10] H. Daub *et al.*, "Kinase-selective enrichment enables quantitative phosphoproteomics of the kinome across the cell cycle," *Mol Cell,* vol. 31, no. 3, pp. 438-48, Aug 8 2008, doi: 10.1016/j.molcel.2008.07.007.

[11] S. K. Hanks and A. M. Quinn, "Protein kinase catalytic domain sequence database: identification of conserved features of primary structure and classification of family members," *Methods Enzymol,* vol. 200, pp. 38-62, 1991, doi: 10.1016/0076-6879(91)00126-h.

[12] W. Kabsch, "Integration, scaling, space-group assignment and post-refinement," *Acta Crystallogr D Biol Crystallogr,* vol. 66, no. Pt 2, pp. 133-44, Feb 2010, doi: 10.1107/S0907444909047374.

[13] S. Mathea *et al.*, "Conformational plasticity of the ULK3 kinase domain," *Biochem J,* vol. 478, no. 14, pp. 2811-2823, Jul 30 2021, doi: 10.1042/BCJ20210257.

[14] P. Emsley, B. Lohkamp, W. G. Scott, and K. Cowtan, "Features and development of Coot," *Acta Crystallogr D Biol Crystallogr,* vol. 66, no. Pt 4, pp. 486-501, Apr 2010, doi: 10.1107/S0907444910007493.

[15] T. Anastassiadis, S. W. Deacon, K. Devarajan, H. Ma, and J. R. Peterson, "Comprehensive assay of kinase catalytic activity reveals features of kinase inhibitor selectivity," *Nat Biotechnol,* vol. 29, no. 11, pp. 1039-45, Oct 30 2011, doi: 10.1038/nbt.2017.

[16] M. Tauro *et al.*, "Bone-Seeking Matrix Metalloproteinase-2 Inhibitors Prevent Bone Metastatic Breast Cancer Growth," *Mol Cancer Ther,* vol. 16, no. 3, pp. 494-505, Mar 2017, doi: 10.1158/1535-7163.MCT-16-0315-T.

[17] C. A. Schneider, W. S. Rasband, and K. W. Eliceiri, "NIH Image to ImageJ: 25 years of image analysis," *Nat Methods,* vol. 9, no. 7, pp. 671-5, Jul 2012, doi: 10.1038/nmeth.2089.

[18] Y. Kim, M. D. Brodt, S. Y. Tang, and M. J. Silva, "MicroCT for Scanning and Analysis of Mouse Bones," *Methods Mol Biol,* vol. 2230, pp. 169-198, 2021, doi: 10.1007/978-1-0716-1028-2_11.

[19] A. Silva, T. Jacobson, M. Meads, A. Distler, and K. Shain, "An Organotypic High Throughput System for Characterization of Drug Sensitivity of Primary Multiple Myeloma Cells," (in English), *Jove-J Vis Exp,* no. 101, Jul 2015, doi: ARTN e53070
10.3791/53070.

[20] P. Sudalagunta *et al.*, "A pharmacodynamic model of clinical synergy in multiple myeloma," *EBioMedicine,* vol. 54, p. 102716, Apr 2020, doi: 10.1016/j.ebiom.2020.102716.

[21] A. G. M. Mostofa *et al.*, "Plasma cell dependence on histone/protein deacetylase 11 reveals a therapeutic target in multiple myeloma," *JCI Insight,* vol. 6, no. 24, 12/22/ 2021, doi: 10.1172/jci.insight.151713.

[22] Y. Ren *et al.*, "PLK1 stabilizes a MYC-dependent kinase network in aggressive B cell lymphomas," *The Journal of Clinical Investigation,* vol. 128, no. 12, pp. 5517-5530, 12/03/ 2018, doi: 10.1172/JCI122533.

[23] A. Silva *et al.*, "An Ex Vivo Platform for the Prediction of Clinical Response in Multiple Myeloma," *Cancer Research,* vol. 77, no. 12, pp. 3336-3351, 2017, doi: 10.1158/0008-5472.CAN-17-0502.

[24] X. Zhao *et al.*, "BCL2 Amplicon Loss and Transcriptional Remodeling Drives ABT-199 Resistance in B Cell Lymphoma Models," *Cancer Cell,* vol. 35, no. 5, pp. 752-766.e9, 2019, doi: 10.1016/j.ccell.2019.04.005.

[25] L. Zhou *et al.*, "IAP and HDAC inhibitors interact synergistically in myeloma cells through noncanonical NF-κB– and caspase-8–dependent mechanisms," *Blood Advances,* vol. 5, no. 19, pp. 3776-3788, 2021, doi: 10.1182/bloodadvances.2020003597.
